# Digital quantification of somatostatin receptor subtype 2a immunostaining: a validation study

**DOI:** 10.1530/EJE-22-0339

**Published:** 2022-06-30

**Authors:** Claudia Campana, Peter M van Koetsveld, Richard A Feelders, Wouter W de Herder, Anand M Iyer, Marie-Louise F van Velthuysen, Marije J Veenstra, Elisabeth S R van den Dungen, Sanne E Franck, Diego Ferone, Federico Gatto, Leo J Hofland

**Affiliations:** 1 Division of Endocrinology, Department of Internal Medicine, Erasmus Medical Center, Rotterdam, The Netherlands; 2 Endocrinology Unit, Department of Internal Medicine and Medical Specialties, School of Medical and Pharmaceutical Sciences, University of Genova, Genova, Italy; 3 Department of Pathology, Erasmus MC Cancer Institute, University Medical Center Rotterdam, Rotterdam, The Netherlands; 4 Endocrinology Unit, IRCCS Ospedale Policlinico San Martino, Genova, Italy

## Abstract

**Objective:**

The aim of this study was to develop an open-source and reproducible digital quantitative analysis (DIA) of somatostatin receptor subtype 2a (SST_2_) staining in formalin-fixed paraffin-embedded tissues of pancreatic neuroendocrine tumors (panNETs) and growth hormone (GH)-secreting pituitary adenomas (GHomas).

**Design:**

SST_2_ immunostaining of 18 panNETs and 39 GHomas was assessed using a novel DIA protocol and compared with a widely used semi-quantitative immunoreactivity score (IRS).

**Methods:**

The DIA software calculates the staining intensity/area and the percentage of positive cells (%PC). Four representative images were selected for each sample by two independent selectors (S_1_ and S_2_), with the analysis performed by two independent analyzers (A_1_ and A_2_). Agreement between observers was calculated using the concordance correlation coefficient (CCC).

**Results:**

In panNETs, the CCC ranged 0.935–0.977 for intensity/area and 0.942–0.983 for %PC. In GHomas, the CCC ranged 0.963–0.997 for intensity/area and 0.979–0.990 for %PC. In both panNETs and GHomas, the DIA staining intensity was strongly correlated with the IRS (Spearman rho: 0.916–0.969, *P* < 0.001), as well as the DIA %PC with the IRS %PC (Spearman rh: 0.826–0.881, *P* < 0.001). In GHomas, the biochemical response to somatostatin receptor ligands correlated with SST_2_ expression, evaluated both as DIA intensity/area (Spearman rho: −0.448 to −0.527, *P* = 0.007–0.004) and DIA %PC (Spearman rho: −0.558 to −0.644, *P* ≤ 0.001).

**Conclusions:**

The DIA has an excellent inter-observer agreement and showed a strong correlation with the widely used semi-quantitative IRS. The DIA protocol is an open-source, highly reproducible tool and provides a reliable quantitative evaluation of SST_2_ immunohistochemistry.

## Introduction

Somatostatin receptors (SSTs) belong to a family of G-protein-coupled receptors widely expressed in the endocrine system and play a crucial role in the regulation of hormonal secretion and cell growth (1). Five different SST subtypes have been identified. Among these, the subtype 2 (SST_2_) represents the main target of first-generation somatostatin receptor ligands (fg-SRLs) (1). Tumors arising from the endocrine system mostly retain SST expression,and therefore, fg-SRLs represent a valuable treatment option (2, 3).

Fg-SRLs are widely used in the management of neuroendocrine tumors (NETs) to control hormonal hypersecretion (if any) and tumor growth (4, 5). A positive SST_2_ immunostaining is a favorable independent prognostic factor in NETs (6, 7, 8), and a correlation between SST_2_ expression assessed by immunohistochemistry and functional imaging has been demonstrated as well (6, 9).

Moreover, fg-SRLs represent the first-line medical treatment for GH-secreting pituitary adenomas (GHomas) (10). Several studies have evaluated the clinical, radiological and molecular factors that are able to predict the response to fg-SRLs (11). As expected, the expression of SST_2_ evaluated by immunohistochemistry has a well-recognized role (12, 13). In this light, the 2017 WHO classification proposed a routine evaluation of SST_2_ immunostaining on paraffin-embedded tissues of GHomas. However, the subsequent Consensus Statements on acromegaly management reiterated its investigational role, due to lack of validation and harmonization among the different scoring systems reported in the literature (14, 15). Multiple methods have been proposed to assess SST_2_ immunostaining, but no consensus has been reached, yet. Furthermore, the scoring systems currently in use rely on the subjective estimation of staining intensity and/or percentage of positive cells and, most importantly, are all expressed as semi-quantitative scales (16).

Here, we propose an open-source, novel digital quantification of SST_2_ immunostaining performed on paraffin-embedded tissues by use of standard immunohistochemistry. We investigated the reproducibility of the method evaluating the agreement between different operators. Finally, we compared the results of the digital analysis with the widely used immunoreactivity score (IRS), which has been routinely used in our laboratory in the last 10 years (17, 18, 19, 20, 21).

## Patients and methods

### Patients

Eighteen patients diagnosed for pancreatic NETs (panNETs), who underwent surgical resection of the primary tumor between 2004 and 2012 at the Erasmus Medical Center, were analyzed. Furthermore, 39 pituitary tumors from patients diagnosed with acromegaly, who underwent neurosurgery at our Institution, were evaluated. Data regarding patients’ clinical characteristics, medical treatment before surgery and disease control (reported as age-adjusted IGF-1 values, normalized to the upper limit of normality (ULN)) were already described in a previous publication from our group (17).

A detailed description of patients’ characteristics is reported in Table 1 and in the Results section.

**Table 1:** General and clinical characteristics of patients diagnosed with panNET or acromegaly included in the study. Data are presented as *n* (%) or as mean± s.d.

Characteristics	Values
panNET	
Females	10 (56)
Males	8 (44)
Age^a^ (years)	52.9 ± 11.9
Stage	
I	2 (11)
II	6 (33)
III	2 (11)
IV	8 (44)
Grading	
G1	12 (67)
G2	5 (28)
G3	1 (6)
Hormonal secretion	
Non-secreting pNET	12 (67)
Insulin	4 (22)
Gastrin	1 (6)
Glucagon	1 (6)
Treatment before surgery^b^	
None	6 (33)
PRRT	5 (28)
Chronic SRLs administration^c^	4 (22)
Perioperative SRLs administration	5 (28)
GH-secreting pituitary adenomas	
Females	15 (40)
Males	23 (60)
Age^d^ (years)	40.8 ± 12.2
Microadenoma	2 (5)
Macroadenoma	36 (95)
Medical treatment before surgery	
Naïve	23 (60.5)
SRLs	8 (21.1)
SRLs+PEGV	7 (18.4)

^a^Age at time of diagnosis; ^b^Three patients received multiple treatments (one PRRT and long-term SRL therapy; one long-term SRL therapy and perioperative SRL administration, and another one PRRT and perioperative SRL administration); no information on treatment before surgery in one patient; ^c^Continued until the surgery; ^d^Age at time of surgery

PRRT, peptide receptor radionuclide therapy; SRL, somatostatin receptor ligand.

Permission from the Institutional Review Board of the Erasmus MC was obtained. The study was performed retrospectively and according to the guidelines of the Central Committee on Research involving Human Subjects.

### Immunohistochemistry

For panNET samples, SST_2_ immunostaining was performed using a manual protocol, as previously described (19). The anti-SST_2_ rabbit monoclonal antibody (MAB) (Biotrend, Köln, Germany) was used at a dilution of 1:25.

The GHoma tissue samples included in the current validation study were previously stained for SST_2_ using a fully automated method (Ventana BenchMark ULTRA stainer, Tucson, Arizona, USA) and scored by use of the semi-quantitative IRS method, as already reported by Franck and colleagues (17). The anti-SST_2_ rabbit MAB (BioTrend) was used at a dilution of 1:25. One patient of the original cohort was excluded due to the low quality of the hematoxylin staining.

SST_2_ staining was quantified using both the IRS and the novel digital image analysis (DIA) method. For the DIA, the tissue slides were digitalized using the NanoZoomer 2.0 HT (Hamamatsu, Naka-ku, Hamamatsu City, Japan).

### Immunoreactivity score

The IRS was performed as previously described (19, 22). Briefly, two investigators independently scored the percentage of positive-stained cells (%PC) from 0 to 4 (0: no positive cells; 1: <10%; 2: 10–50%; 3: 51–80%; 4: >80%) and the staining intensity from 0 to 3 (0: no staining; 1: weak staining; 2: moderate staining; 3: strong staining). The IRS was then calculated as the product of these two scores (range, 0–12).

### Digital image analysis

The DIA was performed with the open-source software Cellprofiler version 4.0.7 (the pipeline can be freely downloaded from https://cellprofiler.org/published-pipelines) (23).

**Figure 1:**
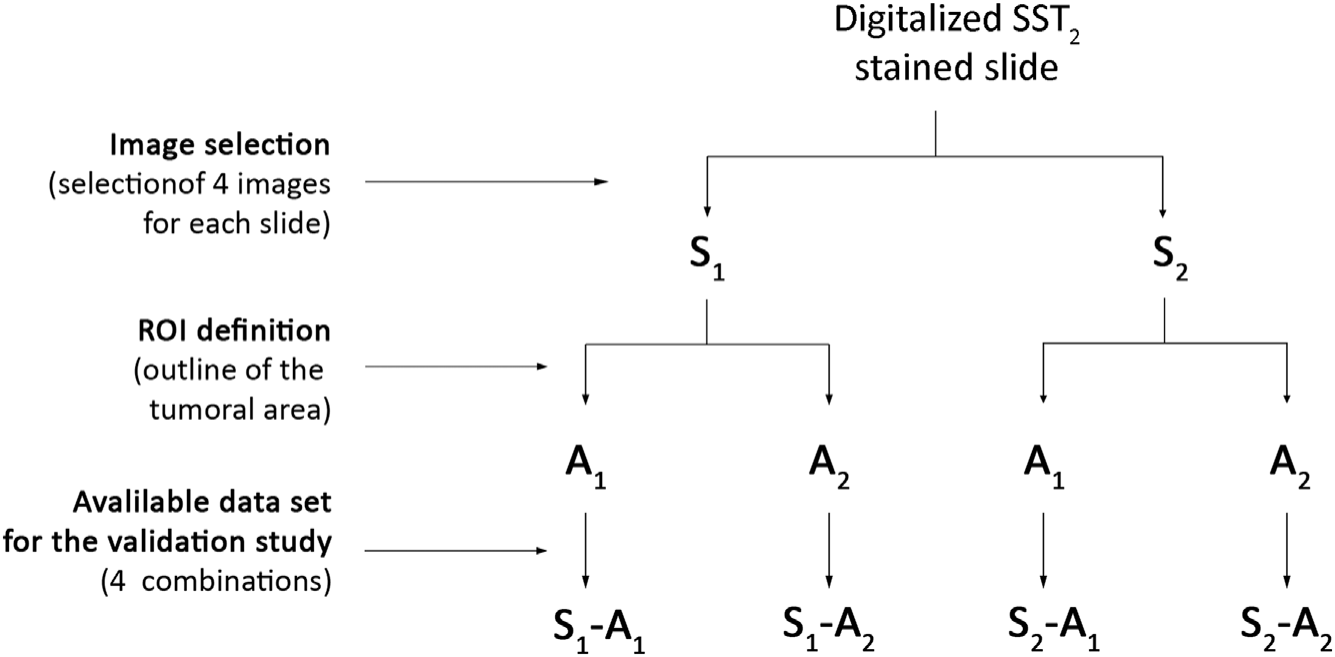
Outline of the validation study. When the tissue was too small to allow the selection of four different images, the maximum possible number of images was acquired. SST_2_, somatostatin receptor subtype 2; ROI, region of interest; S_1_, selector 1; S_2_, selector 2; A_1_, analyzer 1; A_2_, analyzer 2.

**Figure 2:**
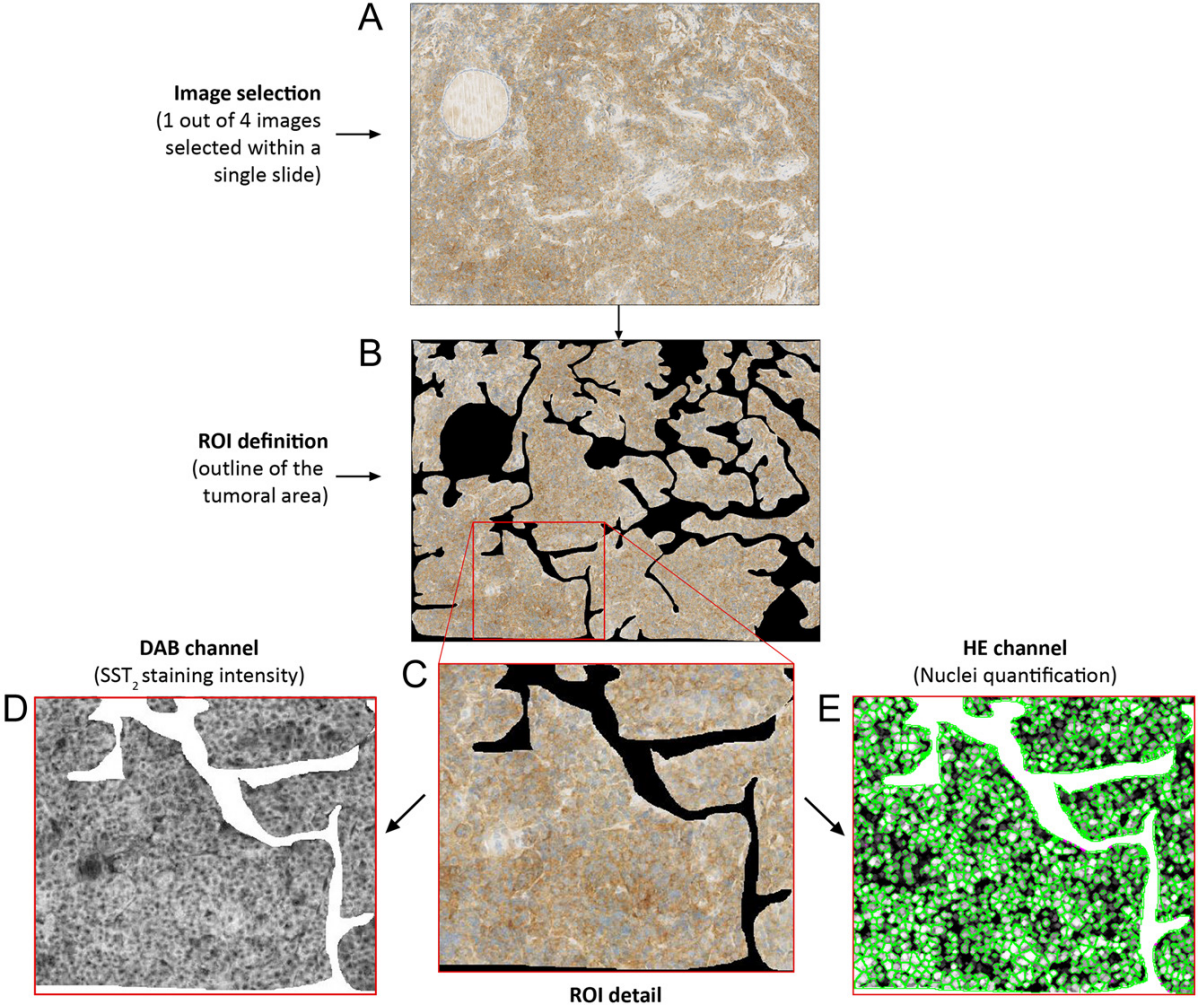
Schematic representation of the digital analysis. Panel A: image identified by the selector (10× magnification). Panel B: region of interest (ROI) definition by the analyzer, consisting in the manual outlining of the tumor tissue with the exclusion of fibrotic tissue, vascular structures and all potential staining artifacts that may impact on SST_2_ quantification. Panel C: higher magnification of Panel B. Panel D: magnification of the grayscale image produced by the software based on 3,3′-diaminobenzidine (DAB) staining (the areas with the weaker staining correspond to the darker areas on the grayscale image). Panel E: magnification of grayscale image produced by the software, based on the hematoxylin staining (the stained objects (nuclei) are converted to white and used for counting the nuclei) and automatic cell delimitation (green lines).

Before starting the software analysis, two manual steps were performed (Fig. 1): (i) independent selection of four representative images (including positive and negative areas, when applicable) for each slide by two investigators, hereafter defined as selector S_1_ and S_2_; (ii) independent definition of the region of interest (ROI; outline of the tumoral area) for each image by two investigators, hereafter defined as analyzer A_1_ and A_2_ (Fig. 2B).

Then, the DIA software converted the images into grayscale pictures, according to the selected staining method (Fig. 2D, E and Supplementary Fig. 1, see section on supplementary materials given at the end of this article) and quantified: number of cells, number of stained cells, intensity of the staining (total intensity: sum of all pixel intensity values) and ROI area (number of analyzed pixels). Finally, two measures were computed: %PC (calculated as (number of stained cells/total number of cells) × 100) and intensity/area (total intensity/ROI area). The intensity/area values ranged between 0 (no staining) and 1 (maximum staining) arbitrary units/pixel. For these two variables, the value representing the measure of a single sample was calculated as the mean of each set of four images analyzed. Additionally, the mean of the measurements of the different selector/analyzer combinations was calculated.

To validate the DIA, we compared this novel method with the widely used IRS. In particular, the intensity/area was correlated with the ‘total’ IRS because both measures consider the staining intensity (DIA: total intensity; IRS: intensity score component) together with a parameter reflecting the distribution of the staining (DIA: ROI area, including both DAB positive and negative pixels; IRS: %PC). Similarly, we correlated the DIA percentage of positive cells with the corresponding component of the IRS.

### Statistical analysis

Categorical data are presented as frequencies and percentages, while quantitative data are reported as mean ± S.D. or as median and interquartile range (IQR) where appropriate. The agreement between selectors/analyzers was assessed by use of the Kruskal–Wallis test (or one-way ANOVA, where appropriate), as well as the concordance correlation coefficient (CCC). The CCC ranges between −1 and 1, where −1 represents complete disagreement, 0 absence of agreement, and 1 complete agreement. The complete agreement can only be obtained if the two vectors are identical. As previously reported, we considered absence of agreement a CCC lower than 0.800, acceptable agreement when the CCC was equal or higher than 0.800 and strong agreement a CCC equal or higher than 0.950 (24). The validation of the DIA with respect to the IRS and the association of the DIA with the biochemical data of the GHomas were performed using the Spearman’s correlation coefficient. Assessment of the predictive discrimination of SST_2_ expression to IGF-1 normalization during treatment with fg-SRLs, evaluated both with DIA and IRS, was performed using the receiver-operating characteristic (ROC) curve. The best-fitting cut-offs were then computed using the Youden index.

Statistical evaluation was performed using GraphPad Prism version 5.01 (GraphPad Software) and the R software, version 4.0.4. Differences were considered statistically significant at *P* < 0.05.

## Results

### Pancreatic neuroendocrine tumors

Patient characteristics are presented in Table 1. Briefly, ten patients (56%) were females, and the mean age at diagnosis was 52.9 ± 11.9 years. Most patients were stage II (*n* = 6, 33%) and stage IV (*n* = 8, 44%). Most of panNETs had a low grade tumor (G1 *n*  = 12, 67%). The majority of panNETs included in our study did not show hormonal hypersecretion (*n* = 12, 67%), although our cohort included also four insulinomas, one gastrinoma and one glucagonoma. Six patients (33%) were naïve to previous treatments at time of surgery, four cases (22%) received fg-SRL therapy before surgery, while neoadjuvant PRRT was administered in five cases (28%).

**Figure 3:**
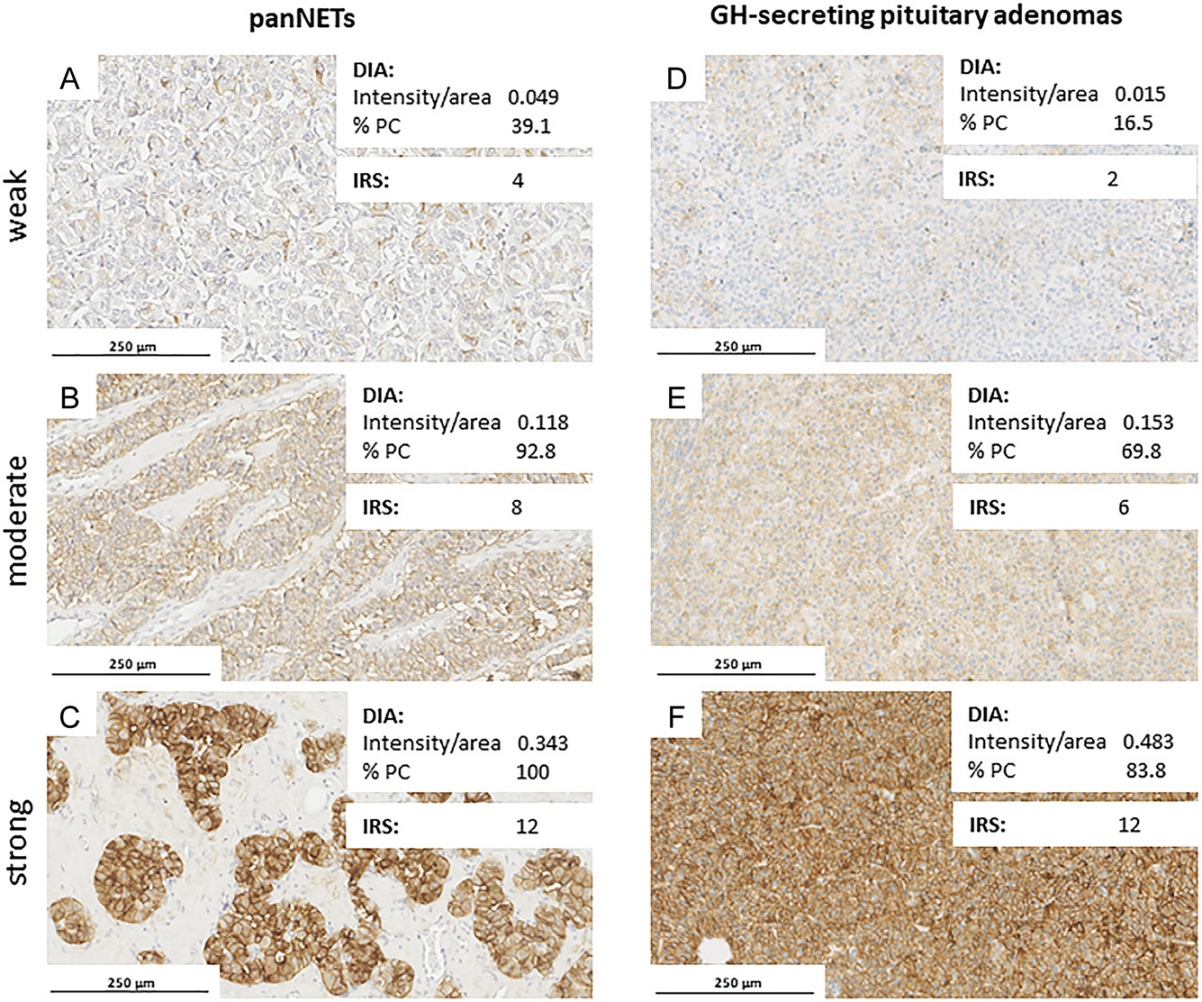
Representative images of SST_2_ expression in panNETs and GH-secreting pituitary adenomas. Intensity/area and percentage of positive cells (%PC) are expressed as the mean of the different selectors/analyzers combinations. Photos were performed at 20× magnification.

**Figure 4:**
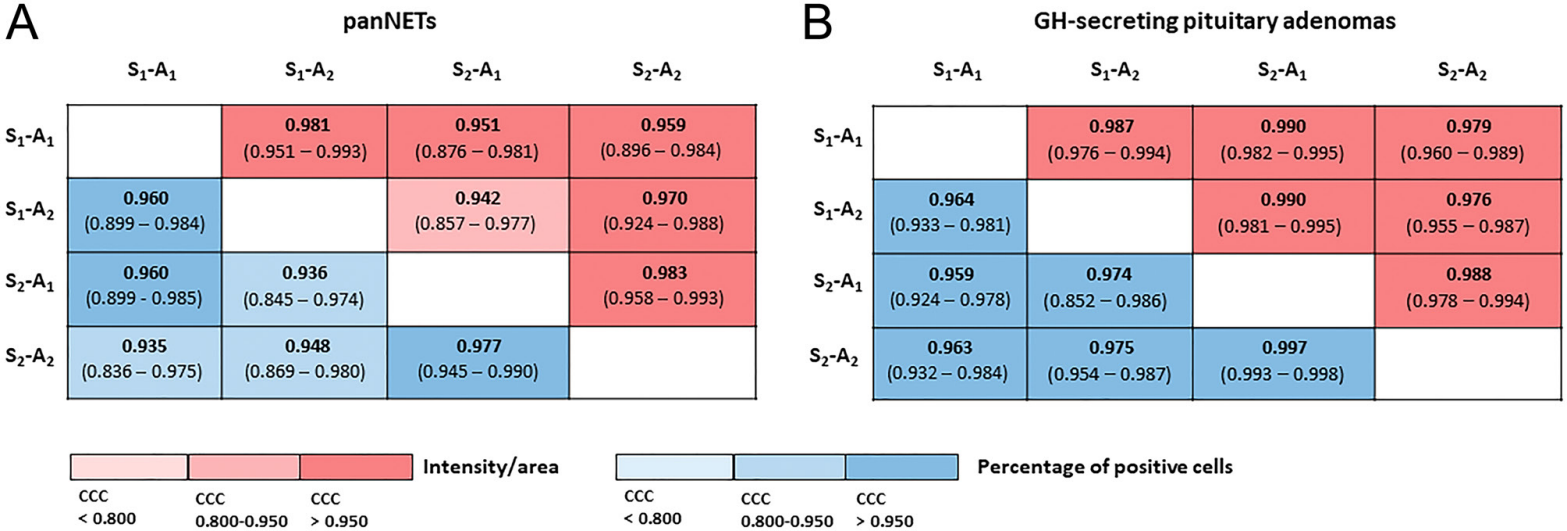
Detailed representation of the concordance correlation coefficients between the different combination of selectors/analyzers in panNETs (panel A) and GH-secreting pituitary adenomas (panel B).

The median SST_2_ IRS was 5 out of the maximal score of 12 (IQR: 1.5–8.0), with a median IRS %PC of 3.5 out of 4 (IQR: 1.25–4.0) (Fig. 3 and Table 2). DIA of the different samples resulted in a median %PC between 40.4 and 49.8%, while the median intensity/area varied between 0.051 and 0.077 among the different combinations of selectors/analyzers (Fig. 3 and Table 2). The CCC between selectors/analyzers for DIA ranged between 0.935 –and 0.977 for the percentage of positive cells and between 0.942 and 0.983 for the intensity/area (Fig. 4A). The agreement between the independent selectors (S_1_ vs S_2_) was generally acceptable for the percentage of positive cells, whereas it was strong for the intensity/area (all but one case: S_1_-A_2_ vs S_2_-A_1_, CCC 0.942; CI 0.857–0.977). Considering the same selector, concordance between different analyzers (A_1_ vs A_2_) was strong in all combinations for both the percentage of positive cells and the intensity/area values (Fig. 4A and Supplementary Figs 2, 3).

**Table 2:** Median values of the digital image analysis (DIA) and the immunoreactivity score (IRS).

	S_1_-A_1_	S_2_-A_1_	S_1_-A_2_	S_2_-A_2_	*P* value	IRS
panNET						
Intensity/area						
Median	0.051	0.066	0.058	0.077	0.812	5.0
IQR	0.010–0.124	0.006–0.107	0.013–0.140	0.014–0.122		1.5–8.0
% positive cells						
Median	40.4	45.8	44.1	49.8	0.989	3.5
IQR	19.3–73.1	15.1–81.8	21.9–76.1	22.5–76.7		1.25–4.0
IRS intensity						
Median						2.0
IQR						1.0–2.0
GH-secreting adenomas						
Intensity/area						
Median	0.120	0.114	0.117	0.120	0.993	6.0
IQR	0.025–0.270	0.017–0.279	0.030–0.273	0.017–0.283		2.5–12.0
% positive cells						
Median	59.5	64.2	61.9	63.1	0.855	3.0
IQR	24.2–76.0	22.3–78.6	15.4–77.9	22.1–78.5		1.5–4.0
IRS intensity						
Median						2.0
IQR						1.5–3.0

*P* values for differences between different selector/analyzers were calculated with the Kruskal– Wallis test (or one-way ANOVA where appropriate).

A_1_, analyzer 1; A_2_, analyzer 2; IRS, immunoreactivity score; IQR, interquartile range;S_1_, selector 1; S_2_, selector 2.

### Correlation between DIA and IRS in panNETs

**Figure 5:**
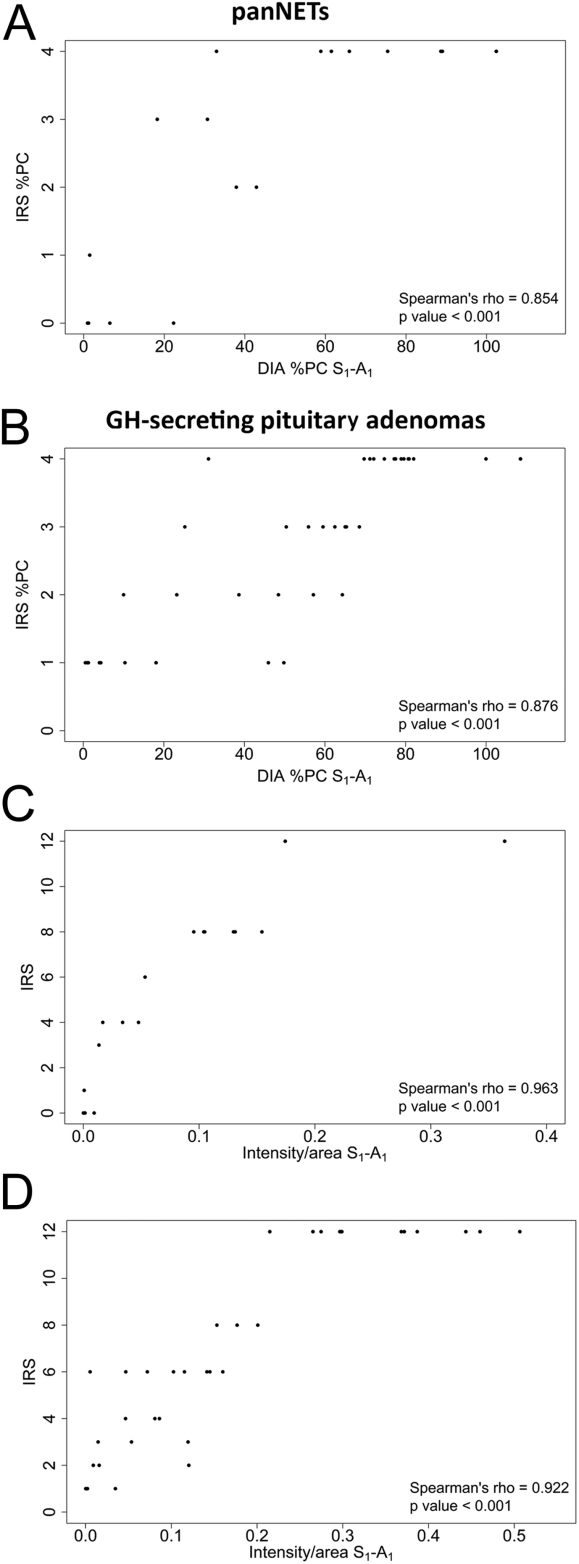
Representative images of the positive correlation between the percentage of positive cells component of the IRS and the DIA’s percentage of positive cells according to S_1_-A_1_ in panNETs (panel A); the percentage of positive cells component of the IRS and the DIA’s percentage of positive cells according to S_1_-A_1_ in GH-secreting adenomas (panel B); the IRS and the DIA’s intensity/area according to S_1_-A_1_ in panNET (panel C) and the IRS and the DIA’s intensity/area according to S_1_-A_1_ in GH-secreting adenomas (panel D). IRS, immunoreactivity score; DIA, digital image analysis; %PC, percentage of positive cells.

The DIA results showed a strong positive correlation with both the IRS %PC and the total IRS (Table 3). The correlations between DIA and IRS for the S_1_-A_1_ analysis are depicted in Fig. 5A and C as a representative evaluation, while the results of the other combinations are reported in Supplementary Fig. 4. The Spearman’s rho for the correlation between the DIA %PC and the IRS %PC value ranged between 0.826 and 0.874 for each selector/analyzer combination (*P* < 0.001 for all). Moreover, the correlation coefficient computed using the mean of the measurements of the different selector/analyzer combinations was comparable to that obtained from the single evaluations (Spearman’s rho, 0.881; *P* < 0.001, Table 3).

**Table 3:** Spearman’s correlation coefficients between DIA and IRS in panNETs.

	Total intensity/area vs IRS	*P* value	Percentage of positive cells vs IRS percentage of positive cells	*P* value
S_1_-A_1_	0.963	<0.001	0.854	<0.001
S_2_-A_1_	0.916	<0.001	0.826	<0.001
S_1_-A_2_	0.935	<0.001	0.874	<0.001
S_2_-A_2_	0.945	<0.001	0.873	<0.001
Mean of all selector/analyzer combinations	0.969	<0.001	0.881	<0.001

A_1_, analyzer 1; A_2_, analyzer 2; DIA, digital image analysis; IRS, immunoreactivity score; Mean of all selector/analyzer combinations: mean of S_1_-A_1_, S_1_-A_2_, S_2_-A_1_ and S_2_-A_2_ values; S_1_, selector 1; S_2_, selector 2.

As expected, the evaluation of the percentage of positive cells in the tumor tissues was not completely overlapping between DIA and IRS. Two samples that were categorized as negative by the IRS %PC showed about 20% of positive cells by the DIA (with a very low median value of the intensity/area of 0.007). On the other hand, the majority of the samples categorized as highly positive by the IRS %PC (score 4: >80% positive cells) were also highly positive on the DIA analysis (median value of 80.7%) (Fig. 5A and Supplementary Fig. 4B, D, F, H).

The DIA intensity/area and the total IRS showed a very strong correlation for each selector/analyzer combination (Spearman’s rho ranged between 0.916 and 0.963, *P* < 0.001). Again, the mean of the measurements of the different selector/analyzer combinations showed a correlation coefficient comparable to those obtained with the single evaluations (Spearman’s rho, 0.969; *P* < 0.001, Table 3). Noteworthy, the DIA allowed a better discrimination of the different samples included in the same IRS category. Particularly, the two patients classified as IRS 12 showed a clear difference in the DIA intensity/area (values ranging from 0.174 to 0.364; Fig. 5C and Supplementary Fig. 4A, C, E, G).

### GH-secreting pituitary adenomas

Patient characteristics are reported in Table 1. Briefly, 15 patients (40%) were females, the mean age at surgery was 40.8 ± 12.2 years and the vast majority (*n* = 36, 95%) had a macroadenoma at the time of diagnosis. Most of patients (*n* = 23, 60.5 %) were naïve to medical treatment, while eight subjects (21%) were treated with fg-SRL monotherapy and the remaining seven patients (18%) were treated with fg-SRLs in combination with the GH receptor antagonist pegvisomant before surgery (17).

The median SST_2_ IRS was 6.0 out of 12 (IQR, 2.5–12.0), with a median %PC of 3.0 out of 4 (IQR, 1.5–4.0) (Fig. 3 and Table 2). For DIA, the median %PC in the different samples ranged between 59.5 and 64.2%, while the median intensity/area varied between 0.114 and 0.120 among the different combinations of selectors/analyzers (Fig. 3 and Table 2). No significant association of SST_2_ expression was observed with clinical variables such as age, gender and adenoma size. No significant difference was observed between the patients pretreated with fg-SRL monotherapy and those subjects naïve to medical treatment before surgery (both for intensity/area and %PC; data not shown).

As shown in Fig. 4B, the CCC showed a strong agreement between the different analyzer/selector combinations, ranging between 0.959 and 0.997 for the %PC and between 0.976 and 0.990 for the intensity/area (see also Supplementary Figs 5 and 6).

### Correlation between DIA and IRS in GH-secreting pituitary adenomas

Similar to the findings in the panNET cohort, the DIA showed a strong positive correlation with the IRS in GHoma tissues as well (Table 4). The correlations between DIA and IRS for the S_1_-A_1_ analysis are depicted in Fig. 5B and D as a representative evaluation, while the results of the other combinations are reported in Supplementary Fig. 7.

**Table 4:** Spearman’s correlation coefficients between DIA and IRS in GH-secreting pituitary adenomas.

	Total intensity/area vs IRS	*P* value	Percentage of positive cells vs IRS percentage of positive cells	*P* value
S_1_-A_1_	0.921	<0.001	0.876	<0.001
S_2_-A_1_	0.928	<0.001	0.840	<0.001
S_1_-A_2_	0.922	<0.001	0.863	<0.001
S_2_-A_2_	0.928	<0.001	0.844	<0.001
Mean of all selector/analyzer combinations	0.924	<0.001	0.872	<0.001

A_1_, analyzer 1; A_2_, analyzer 2; DIA, digital image analysis; IRS, immunoreactivity score; Mean of all selector/analyzer combinations: mean of S_1_-A_1_, S_1_-A_2_, S_2_-A_1_ and S_2_-A_2_ values; S_1_, selector 1; S_2_, selector 2.

The Spearman’s rho for the correlation between DIA %PC and the corresponding IRS %PC value ranged between 0.840 and 0.876 for each selector/analyzer combination (*P* < 0.001 for all).

Despite the strong correlations observed, the tissues categorized by the IRS %PC as score 2 (20–50 % of positive cells) and score 3 (50–80% of positive cells) showed a considerable overlap in terms of DIA %PC values. On the other hand, the ‘extremes’ (IRS %PC score 1 (<20% positive cells) and IRS %PC score 4 (>80% positive cells)) were almost perfectly discriminated by the DIA analysis (Fig. 5B and Supplementary Fig. 7B, D, F, H).

As observed in the panNET cohort, the DIA intensity/area and the total IRS showed a very strong correlation for each selector/analyzer combination (Spearman’s rho ranged between 0.921 and 0.928, *P* < 0.001). The mean of the measurements of all the different selector/analyzer combinations showed a correlation coefficient comparable to those obtained with the single evaluations (Spearman’s rho, 0.924; *P* < 0.001, Table 4).

The DIA allowed a better discrimination of the different samples included in the same category by use of the IRS. In particular, among those samples classified with a total IRS of 12 (maximum score), we observed a wide range in the DIA intensity/area (values from 0.215 to 0.507; Fig. 5D and Supplementary Fig. 7A, C, E, G).

### Correlations between DIA and biochemical response to fg-SRLs in GH-secreting pituitary adenomas

**Figure 6:**
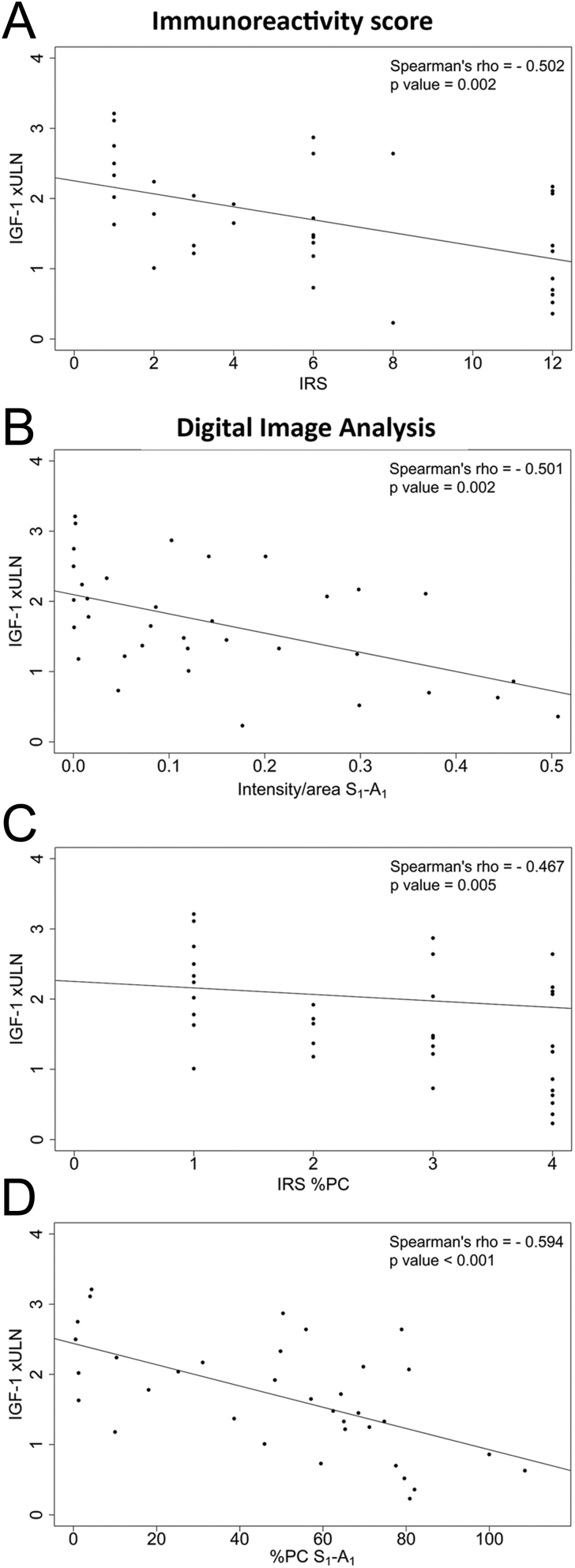
IGF-1 xULN during fg-SRL monotherapy was inversely correlated to SST_2_ staining represented as IRS (panel A), intesity/area according to S_1_-A_1_ (panel B), percentage of positive cell component of the IRS (panel C) and percentage of positive cell according to S_1_-A_1_ (panel D). IGF-1, insulin growth factor 1; ULN, upper limit of normality; IRS, immunoreactivity score; %PC, percentage of positive cells.

The DIA confirmed the negative correlation already observed between SST_2_ IRS and the age-adjusted IGF-1 values reached after long-term fg-SRL treatment (namely, the highest SST_2_ expression and the lowest IGF-1 levels) (17). The correlations obtained by the S_1_-A_1_ analysis are depicted in Fig. 6B and D as a representative evaluation, while the other correlations are reported in Supplementary Fig. 8.

**Figure 7:**
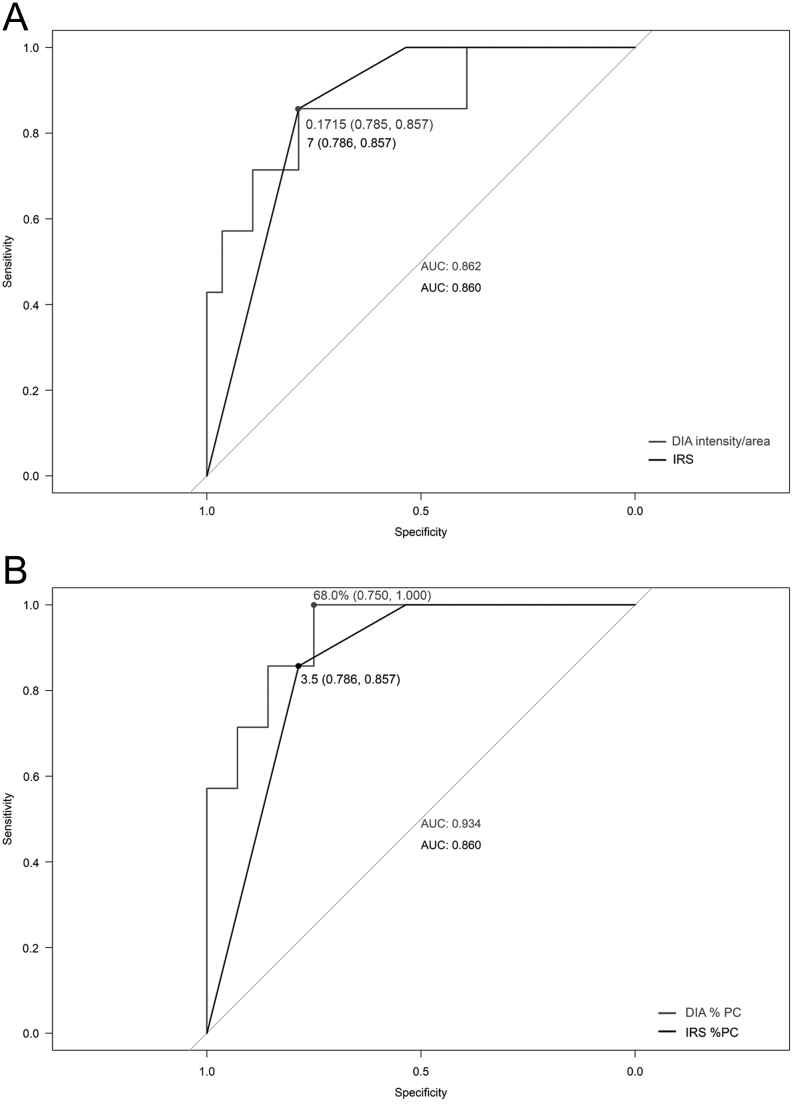
Accuracy of the DIA and IRS for predicting IGF-1 normalization during fg-SRL monotherapy. Panel A shows the ROC curves obtained with DIA intensity/area ( gray line) and IRS (black line); panel B shows the ROC curves obtained with DIA %PC (gray line) and IRS %PC (black line). The dots represent the best-fitting cut-offs. Specificity and sensitivity values are reported in brackets. AUC, area under the curve; IGF-1, insulin growth factor 1; IRS, immunoreactivity score; %PC, percentage of positive cells.

The DIA intensity/area showed a significant negative correlation with IGF-1 values xULN after fg-SRL treatment for each selector/analyzer combination (Spearman’s rho ranged from −0.448 to −0.527, *P* = 0.001–0.007). The mean of the measurements of the different selector/analyzer combinations showed a correlation coefficient comparable to those obtained with the single evaluations (Spearman’s rho, −0.482; *P* = 0.003, Table 5). At ROC curve analysis, performed to evaluate the discrimination of SST_2_ expression on IGF-1 normalization, we observed an AUC between 0.852 (95% CI, 0.679–1) and 0.878 (95% CI, 0.703–1) in the different selector/analyzer combinations. The AUC obtained using the mean of the different measurements was almost superimposable (0.862; 95% CI, 0.689–1), being not significantly different from the analysis obtained with IRS (AUC = 0.860; 95% CI, 0.745–0.975; *P* = 0.963). The best-fitting cut-off predictive of the response to fg-SRLs was 0.1715 intensity/area, showing 86% sensitivity and 79% specificity (Fig. 7A).

**Table 5:** Spearman’s correlation coefficients between DIA and IGF-1 values in acromegaly patients during fg-SRL monotherapy.

	Total intensity/area	*P* value	Percentage of positive cells	*P* value
S_1_-A_1_	−0.501	0.002	−0.594	<0.001
S_2_-A_1_	−0.474	0.004	−0.600	<0.001
S_1_-A_2_	−0.527	0.001	−0.644	<0.001
S_2_-A_2_	−0.448	0.007	−0.588	<0.001
Mean of all selector/analyzer combinations	−0.482	0.003	−0.617	<0.001
IRS	−0.501	0.002		
IRS percentage of positive cells			−0.467	0.005

A_1_, analyzer 1; A_2_, analyzer 2; DIA, digital image analysis; fg-SRLs, first-generation somatostatin receptor ligand; IRS, immunoreactivity score; mean of all selector/analyzer combinations: mean of S_1_-A_1_, S_1_-A_2_, S_2_-A_1_ and S_2_-A_2_ values; S_1_, selector 1; S_2_, selector 2.

Interestingly, when considering the %PC alone, we observed that the correlations between DIA %PC and the IGF-1 values xULN after fg-SRL treatment were stronger (Spearman’s rho ranging from −0.588 to −0.644, *P* < 0.001 for all the selector/analyzer combinations) compared to the one obtained using the IRS %PC (Spearman’s rho, −0.467; *P* = 0.005) (Fig. 6C, D and Table 5). Similarly, the DIA %PC demonstrated a high accuracy in predicting the response to fg-SRLs (AUC ranging between 0.898 (95% CI 0.786–1) and 0.944 (95% CI 0.870–1) in the different selector/analyzer combinations). The ROC curve obtained using the mean of the different measurements was almost superimposable (AUC = 0.934; 95% CI, 0.846–1) and showed a higher accuracy compared to the IRS %PC (AUC = 0.860; 95% CI, 0.752–0.967; *P* = 0.049). The presence of >68% SST_2-_positive cells evaluated with DIA predicted the response to fg-SRL with 100% sensitivity and 75% specificity (Fig. 7B).

## Discussion

In the present study, for the first time, we have performed a quantitative digital evaluation of SST_2_ immunoreactivity in both panNET and GHoma samples, by use of a widely available open-source software.

In order to validate the DIA protocol, we compared the performance of this novel method to the widely used semi-quantitative IRS. The IRS was first established for the quantification of estrogen receptors in breast cancers (22), and it has been subsequently used by different groups for SST_2_ evaluation in both GHomas (19, 25) and NETs (26, 27). SST_2_ IRS has shown a strong correlation with the biochemical response to fg-SRL treatment in GHomas (17, 19, 25), as well as a moderate correlation with the results of functional imaging (namely, 68Ga-DOTA-NOC PET/CT) in NETs (26).

The strong positive correlation that we observed between the IRS and the DIA parameters supports the reliability of the DIA. The DIA performed equally well with respect to IRS in both panNETs and GHomas, despite the immunohistochemistry being performed using different protocols in the two disease groups (manual vs automated, respectively).

Our results highlight DIA as a highly reproducible method, since the CCC observed between the different combinations of selectors/analyzers were overall extremely satisfying. Unlike the currently available scoring systems, which rely on the subjective evaluation of the staining intensity and/or the percentage of positive cells (prone to inter-observer variability and difficult to be standardized), the DIA is by definition less-dependent on subjective estimation (28).

However, we have to acknowledge that our DIA protocol includes two non-automated steps, consisting of manual image selection, which may represent a potential source of bias. The selection of representative tumor areas was necessary because the CellProfiler software does not support analysis of whole slide scans. Since it is known that SST_2_ expression can exhibit intratumoral heterogeneity in both panNETs and GHomas (29, 30), we opted for the selection of four images for each slide as a balance between the complete representation of the tissue slide and the feasibility of the downstream analysis. The data showed an excellent agreement, again demonstrating the reproducibility of the DIA protocol. This was confirmed also for the second step, as CCC showed an excellent agreement between the different analyzers in both panNETs and GHomas, supporting the low impact of the manual ROI definition.

Noteworthy, using the mean of the measurements obtained from the different selectors/analyzers did not significantly improve the correlations observed between DIA and IRS, compared to the use of a single selector/analyzer evaluation. This finding reflects the robustness of the CCC analysis and the reproducibility of the DIA.

In this light, we conclude that the analysis can be performed by a single operator without any major impact on the quality of the results. This could represent a further advantage compared to the routinely used semi-quantitative methods that should be performed by two experienced pathologists, due to their potential high inter- and intra-observer variability.

Interestingly, the DIA showed a stronger discriminatory power compared to the IRS score. Among the tissues classified as IRS 12 (all had the maximum achievable score), the DIA intensity/area provided a wide range of high level values. Indeed, the DIA express the results as a continuous variable with a wide dynamic range, thus allowing a better characterization of the receptor staining, while the IRS has an intrinsic semi-quantitative nature and a more limited dynamic range.

As described above, our group previously reported that SST_2_ IRS correlated with the age-adjusted IGF-1 levels after fg-SRL therapy in the cohort of acromegaly patients we analyzed by DIA in the present study (17). This correlation has been confirmed when evaluating the SST_2_ expression using our novel DIA protocol. Giving the satisfactory sensitivity and specificity shown by the DIA in predicting IGF-1 normalization during fg-SRLs, together with the high reproducibility of the method, this novel analysis can represent an important tool for patients’ selection. Interestingly, while the DIA intensity/area showed a comparable correlation and accuracy to the one previously described with IRS, a stronger correlation and a better accuracy were found using the DIA %PC values. These results suggest that the %PC closely relates to the hormonal response during fg-SRL treatment in GHomas (more than the staining intensity). Therefore, we hypothesize that the use of a continuous variable with a more precise discrimination (intrinsic characteristics of the DIA) allowed us to better define this association compared to the IRS categorical classification. Due to the heterogeneous expression of SST_2_ in GHomas, the proportion of receptor-expressing cells seems to be a key determinant of the overall therapeutic response to fg-SRLs.

Watanabe and colleagues recently reported a quantitative digital analysis evaluation of SST_2_ in panNET and gastro-intestinal (GI) NET tissues, performed using a commercially available software (namely, HALO Membrane) (31, 32). The authors compared the DIA with different semi-quantitative scores (i.e. IRS and Volante score for GI-NETs and HER2 score for panNETs). An overall good concordance between the DIA and the manual semi-quantitative scores was reported in both studies. However, as acknowledged by the authors themselves, the high cost of the software may represent a major limitation of this method. The optimization and validation of an open-source software (e.g. CellProfiler) could make the DIA more affordable and widely available.

Finally, our study has some limitations. Although we demonstrated that the two manual steps of our protocol do not affect the data reproducibility, the DIA evaluation using the CellProfiler software is not completely automated, and the expertise of a skilled pathologist cannot be ruled out when transposing this method into the daily clinical practice. Furthermore, additional studies aimed to assess the value of the DIA protocol in terms of correlations with clinical data are strongly needed, particularly with respect to NET patients. In the present cohort, only a minority of patients had functional imaging (namely, ^111^In-pentetreotide scintigraphy) or SRLs treatment, therefore no definitive conclusion could be drawn. In addition, differently from the HALO Membrane algorithm used by Watanabe and colleagues, we have evaluated the whole-cell staining intensity, considering both membranous and cytoplasmic staining. Although some manual scores in use only evaluate the extent of SST_2_ membranous staining (e.g. Volante score), whether the whole receptor expression needs to be considered (irrespective of the subcellular localization) is still matter of debate. In our opinion, in case of pre-surgical treatment with SRLs, the evaluation of the membranous staining alone could lead to an underestimation of the whole receptor pool of the tumor cells (at least in the setting of GHomas and functioning NETs). However, we are aware that only comparative studies aimed to address this issue, evaluating the best correlate with the clinical outcomes, can provide us a more robust direction.

In conclusion, the DIA protocol showed an excellent agreement between the different operators involved. Furthermore, the DIA showed a strong agreement with the widely used IRS, as well as a good correlation with the biochemical response to fg-SRL treatment in GH-secreting pituitary adenomas.

The DIA has a wide dynamic range and is expressed as a continuous variable, allowing us to perform a more detailed characterization of the receptor staining. Therefore, it can provide a more reliable quantitative evaluation of SST_2_ immunostaining compared to the currently available semi-quantitative methods.

## Supplementary Material

Supplementary Figure 1

Supplementary Figure 2

Supplementary Figure 3

Supplementary Figure 4

Supplementary Figure 5

Supplementary Figure 6

Supplementary Figure 7

Supplementary Figure 8

## Declaration of interest

R A F has received research grants from Strongbridge and Corcept and is a consultant for Recordati. W W H has been a speaker for and participated on advisory boards and received research grants from Novartis and Ipsen. D F has been a speaker for and participated on advisory boards and received research grants from Novartis-AAA, Ipsen, Recordati RD, Camurus and Pfizer. F G has been a speaker for Novartis and has participated on advisory boards of Novartis, AMCo Ltd, and IONIS Pharmaceuticals. The other authors have no conflict of interest to declare.

## Funding

This study did not receive any specific grant from any funding agency in the public, commercial or not-for-profit sector.

## Author contribution statement

F Gatto and L J Hofland contributed equally.

## References

[1] Gunther T Tulipano G Dournaud P Bousquet C Csaba Z Kreienkamp HJ Lupp A Korbonits M Castano JP Wester HJ***et al***. International Union of Basic and Clinical Pharmacology. CV. Somatostatin receptors: structure, function, ligands, and new nomenclature. Pharmacological Reviews 2018 70 763–835. (10.1124/pr.117.015388) 30232095 PMC6148080

[2] Hofland LJ Lamberts SW. The pathophysiological consequences of somatostatin receptor internalization and resistance. Endocrine Reviews 2003 24 28–47. (10.1210/er.2000-0001) 12588807

[3] Gatto F Barbieri F Arvigo M Thellung S Amaru J Albertelli M Ferone D Florio T. Biological and biochemical basis of the differential efficacy of first and second generation somatostatin receptor ligands in neuroendocrine neoplasms. International Journal of Molecular Sciences 2019 20 3940. (10.3390/ijms20163940) PMC6720449 31412614

[4] Pavel M Oberg K Falconi M Krenning EP Sundin A Perren A Berruti A & ESMO Guidelines Committee. Electronic address: clinicalguidelines@esmo.org. Gastroenteropancreatic neuroendocrine neoplasms: ESMO Clinical Practice Guidelines for diagnosis, treatment and follow-up. Annals of Oncology 2020 31 844–860. (10.1016/j.annonc.2020.03.304) 32272208

[5] Pavel M Valle JW Eriksson B Rinke A Caplin M Chen J Costa F Falkerby J Fazio N Gorbounova V Enets consensus guidelines for the standards of care in neuroendocrine neoplasms: systemic therapy – biotherapy and novel targeted agents. Neuroendocrinology 2017 105 266–280. (10.1159/000471880) 28351033

[6] Brunner P Jorg AC Glatz K Bubendorf L Radojewski P Umlauft M Marincek N Spanjol PM Krause T Dumont RA***et al***. The prognostic and predictive value of sstr2-immunohistochemistry and sstr2-targeted imaging in neuroendocrine tumors. European Journal of Nuclear Medicine and Molecular Imaging 2017 44 468–475. (10.1007/s00259-016-3486-2) 27539020

[7] Qian ZR Li T Ter-Minassian M Yang J Chan JA Brais LK Masugi Y Thiaglingam A Brooks N Nishihara R***et al***. Association between somatostatin receptor expression and clinical outcomes in neuroendocrine tumors. Pancreas 2016 45 1386–1393. (10.1097/MPA.0000000000000700) 27622342 PMC5067972

[8] Mehta S de Reuver PR Gill P Andrici J D’Urso L Mittal A Pavlakis N Clarke S Samra JS Gill AJ. Somatostatin receptor SSTR-2a expression is a stronger predictor for survival than Ki-67 in pancreatic neuroendocrine tumors. Medicine 2015 94 e1281. (10.1097/MD.0000000000001281) PMC4616753 26447992

[9] Miederer M Seidl S Buck A Scheidhauer K Wester HJ Schwaiger M Perren A. Correlation of immunohistopathological expression of somatostatin receptor 2 with standardised uptake values in 68Ga-DOTATOC PET/CT. European Journal of Nuclear Medicine and Molecular Imaging 2009 36 48–52. (10.1007/s00259-008-0944-5) 18807033

[10] Giustina A Barkhoudarian G Beckers A Ben-Shlomo A Biermasz N Biller B Boguszewski C Bolanowski M Bollerslev J Bonert V***et al***. Multidisciplinary management of acromegaly: a consensus. Reviews in Endocrine and Metabolic Disorders 2020 21 667–678. (10.1007/s11154-020-09588-z) 32914330 PMC7942783

[11] Corica G Ceraudo M Campana C Nista F Cocchiara F Boschetti M Zona G Criminelli D Ferone D Gatto F. Octreotide-resistant acromegaly: challenges and solutions. Therapeutics and Clinical Risk Management 2020 16 379–391. (10.2147/TCRM.S183360) 32440136 PMC7211320

[12] Brzana J Yedinak CG Gultekin SH Delashaw JB Fleseriu M. Growth hormone granulation pattern and somatostatin receptor subtype 2A correlate with postoperative somatostatin receptor ligand response in acromegaly: a large single center experience. Pituitary 2013 16 490–498. (10.1007/s11102-012-0445-1) 23184260

[13] Fougner SL Casar-Borota O Heck A Berg JP Bollerslev J. Adenoma granulation pattern correlates with clinical variables and effect of somatostatin analogue treatment in a large series of patients with acromegaly. Clinical Endocrinology 2012 76 96–102. (10.1111/j.1365-2265.2011.04163.x) 21722151

[14] Melmed S Bronstein MD Chanson P Klibanski A Casanueva FF Wass JAH Strasburger CJ Luger A Clemmons DR Giustina A. A consensus statement on acromegaly therapeutic outcomes. Nature Reviews: Endocrinology 2018 14 552–561. (10.1038/s41574-018-0058-5) PMC7136157 30050156

[15] Inoshita N Nishioka H. The 2017 WHO classification of pituitary adenoma: overview and comments. Brain Tumor Pathology 2018 35 51–56. (10.1007/s10014-018-0314-3) 29687298

[16] Gatto F Arvigo M Ferone D. Somatostatin receptor expression and patients’ response to targeted medical treatment in pituitary tumors: evidences and controversies. Journal of Endocrinological Investigation 2020 43 1543–1553. (10.1007/s40618-020-01335-0) 32557353

[17] Franck SE Gatto F van der Lely AJ Janssen JAMJL Dallenga AHG Nagtegaal AP Hofland LJ Neggers SJCMM. Somatostatin receptor expression in GH-secreting pituitary adenomas treated with long-acting somatostatin analogues in combination with pegvisomant. Neuroendocrinology 2017 105 44–53. (10.1159/000448429) 27455094 PMC5475231

[18] Muhammad A Coopmans EC Gatto F Franck SE Janssen JAMJL van der Lely AJ Hofland LJ Neggers SJCMM. Pasireotide responsiveness in acromegaly is mainly driven by somatostatin receptor subtype 2 expression. Journal of Clinical Endocrinology and Metabolism 2019 104 915–924. (10.1210/jc.2018-01524) 30346538

[19] Gatto F Feelders RA van der Pas R Kros JM Waaijers M Sprij-Mooij D Neggers SJ van der Lelij AJ Minuto F Lamberts SW***et al***. Immunoreactivity score using an anti-sst2A receptor monoclonal antibody strongly predicts the biochemical response to adjuvant treatment with somatostatin analogs in acromegaly. Journal of Clinical Endocrinology and Metabolism 2013 98 E66–E71. (10.1210/jc.2012-2609) 23118420

[20] Coopmans EC Schneiders JJ El-Sayed N Erler NS Hofland LJ van der Lely AJ Petrossians P Potorac J Muhammad A Neggers SJCMM. T2-signal intensity, sstr expression, and somatostatin analogs efficacy predict response to pasireotide in acromegaly. European Journal of Endocrinology 2020 182 595–605. (10.1530/EJE-19-0840) 32375119

[21] Gatto F Feelders RA Franck SE van Koetsveld PM Dogan F Kros JM Neggers SJCMM van der Lely AJ Lamberts SWJ Ferone D***et al***. In vitro head-to-head comparison Between octreotide and pasireotide in GH-secreting pituitary adenomas. Journal of Clinical Endocrinology and Metabolism 2017 102 2009–2018. (10.1210/jc.2017-00135) 28323931

[22] Remmele W Stegner HE. [Recommendation for uniform definition of an immunoreactive score (IRS) for immunohistochemical estrogen receptor detection (ER-ICA) in breast cancer tissue]. Vorschlag zur einheitlichen Definition eines Immunreaktiven Score (IRS) fur den immunhistochemischen Ostrogenrezeptor-Nachweis (ER-ICA) im Mammakarzinomgewebe. Pathologe 1987 8 138–140. 3303008

[23] Stirling DR Swain-Bowden MJ Lucas AM Carpenter AE Cimini BA Goodman A. CellProfiler 4: improvements in speed, utility and usability. BMC Bioinformatics 2021 22 433. (10.1186/s12859-021-04344-9) PMC8431850 34507520

[24] Crandall JP Fraum TJ Lee M Jiang L Grigsby P Wahl RL. Repeatability of (18)F-FDG PET radiomic features in cervical cancer. Journal of Nuclear Medicine 2021 62 707–715. (10.2967/jnumed.120.247999) 33008931 PMC8844259

[25] Casar-Borota O Heck A Schulz S Nesland JM Ramm-Pettersen J Lekva T Alafuzoff I Bollerslev J. Expression of SSTR2a, but not of SSTRs 1, 3, or 5 in somatotroph adenomas assessed by monoclonal antibodies was reduced by octreotide and correlated with the acute and long-term effects of octreotide. Journal of Clinical Endocrinology and Metabolism 2013 98 E1730–E1739. (10.1210/jc.2013-2145) 24092823

[26] Kaemmerer D Peter L Lupp A Schulz S Sanger J Baum RP Prasad V Hommann M. Comparing of IRS and Her2 as immunohistochemical scoring schemes in gastroenteropancreatic neuroendocrine tumors. International Journal of Clinical and Experimental Pathology 2012 5 187–194. 22558472 PMC3341681

[27] Kajtazi Y Kaemmerer D Sanger J Schulz S Lupp A. Somatostatin and chemokine CXCR4 receptor expression in pancreatic adenocarcinoma relative to pancreatic neuroendocrine tumours. Journal of Cancer Research and Clinical Oncology 2019 145 2481–2493. (10.1007/s00432-019-03011-0) 31451931 PMC11810289

[28] Meyerholz DK Beck AP. Principles and approaches for reproducible scoring of tissue stains in research. Laboratory Investigation 2018 98 844–855. (10.1038/s41374-018-0057-0) 29849125

[29] Feijtel D Doeswijk GN Verkaik NS Haeck JC Chicco D Angotti C Konijnenberg MW de Jong M Nonnekens J. Inter and intra-tumor somatostatin receptor 2 heterogeneity influences peptide receptor radionuclide therapy response. Theranostics 2021 11 491–505. (10.7150/thno.51215) 33391488 PMC7738856

[30] Colao A Auriemma RS Lombardi G Pivonello R. Resistance to somatostatin analogs in acromegaly. Endocrine Reviews 2011 32 247–271. (10.1210/er.2010-0002) 21123741

[31] Watanabe H Ide R Yamazaki Y Fujishima F Kasajima A Yazdani S Tachibana T Motoi F Unno M Sasano H. Quantitative digital image analysis of somatostatin receptor 2 immunohistochemistry in pancreatic neuroendocrine tumors. Medical Molecular Morphology 2021 54 324–336. (10.1007/s00795-021-00294-6) 34247274

[32] Watanabe H Fujishima F Komoto I Imamura M Hijioka S Hara K Yatabe Y Kudo A Masui T Tsuchikawa T***et al***. Somatostatin receptor 2 expression profiles and their correlation with the efficacy of somatostatin analogues in gastrointestinal neuroendocrine tumors. Cancers 2022 14 775. (10.3390/cancers14030775) 35159042 PMC8834049

