# Supplementary material for: Digital quantification of somatostatin receptor subtype 2a immunostaining: a validation study
Source: Eur J Endocrinol. 2022 Jun 30;187(3):399–411. doi: 10.1530/EJE-22-0339 (PMC9346267; doi:10.1530/EJE-22-0339)

Supplementary Figure 1.

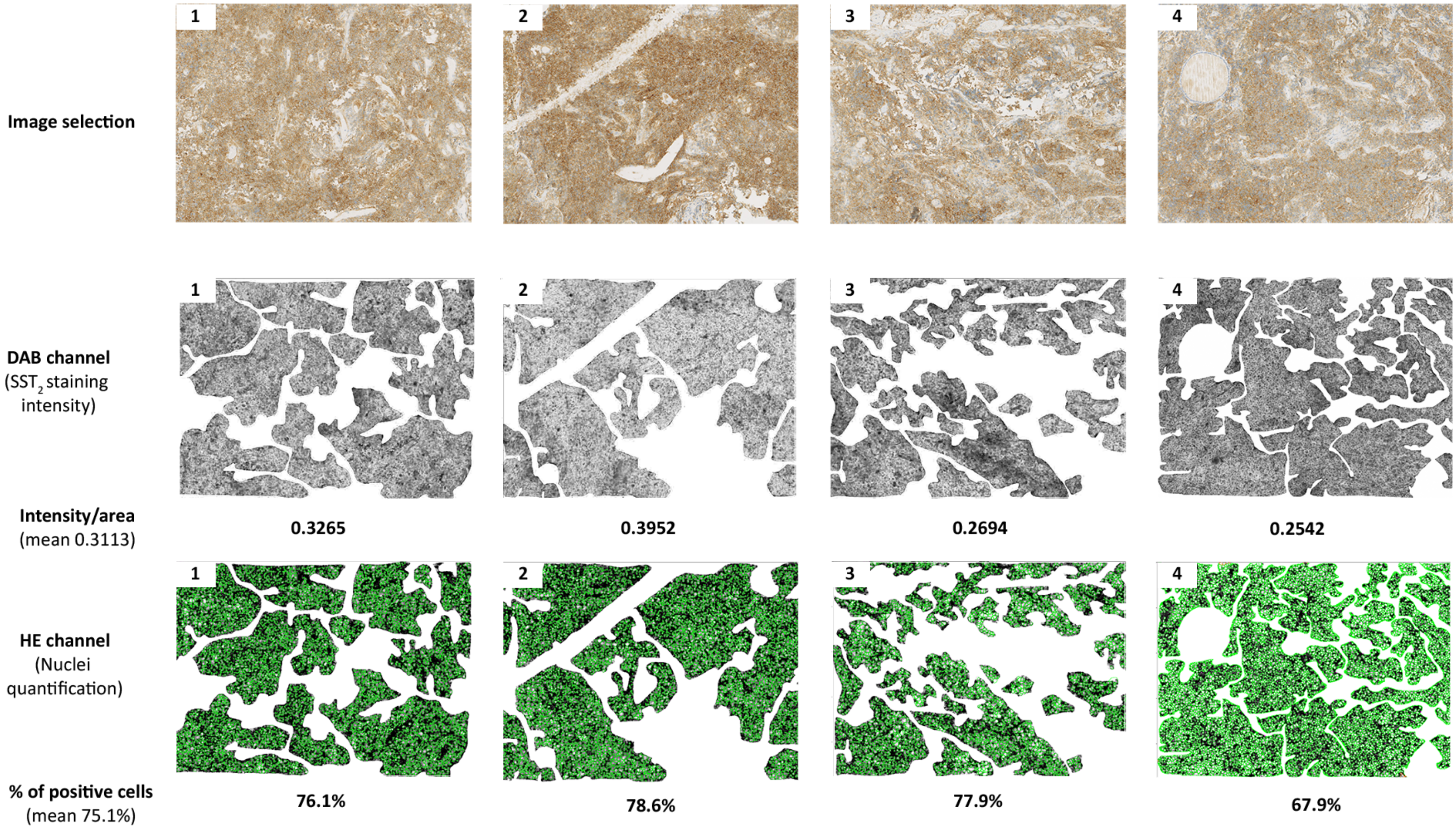

Supplement: Supplementary Figure 1 [file supplementary_figure_1.pdf]
