# Supplementary figures and images for: Digital quantification of somatostatin receptor subtype 2a immunostaining: a validation study
Source: Eur J Endocrinol. 2022 Jun 30;187(3):399–411. doi: 10.1530/EJE-22-0339 (PMC9346267; doi:10.1530/EJE-22-0339)

Supplementary Figure 2.

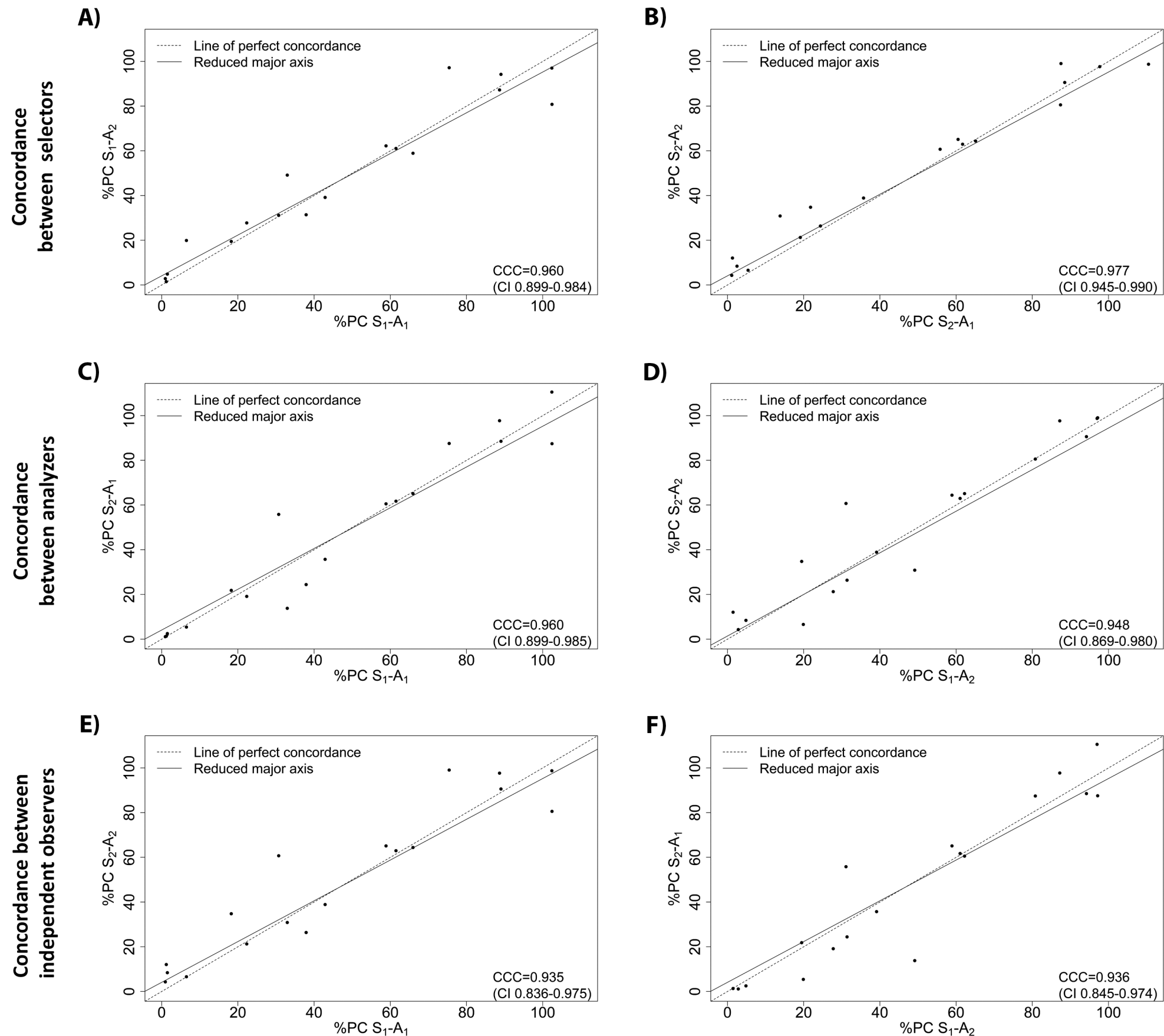

Supplement: Supplementary Figure 2 [file supplementary_figure_2.pdf]

Supplementary Figure 3.

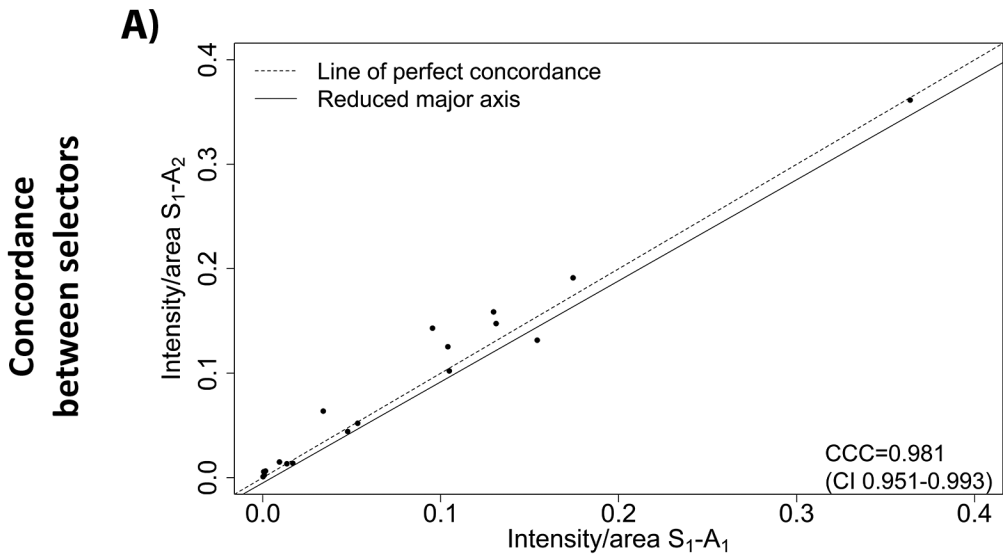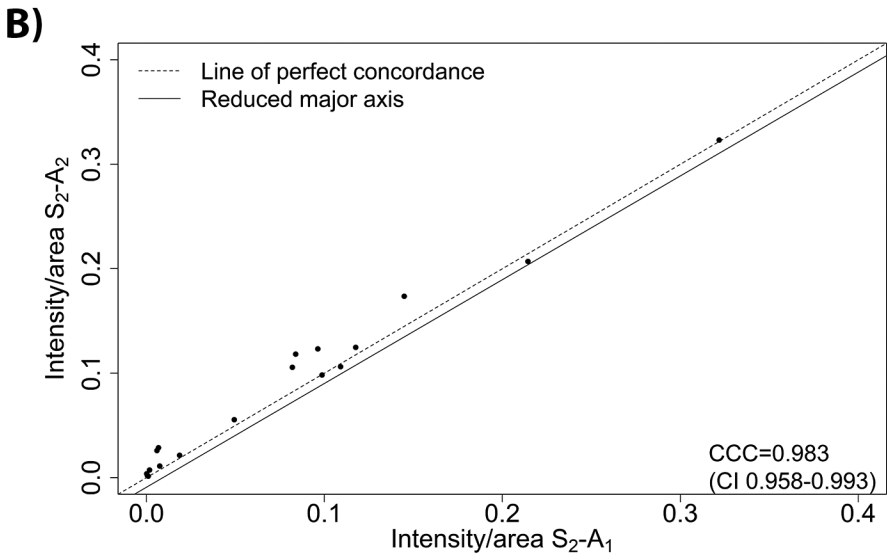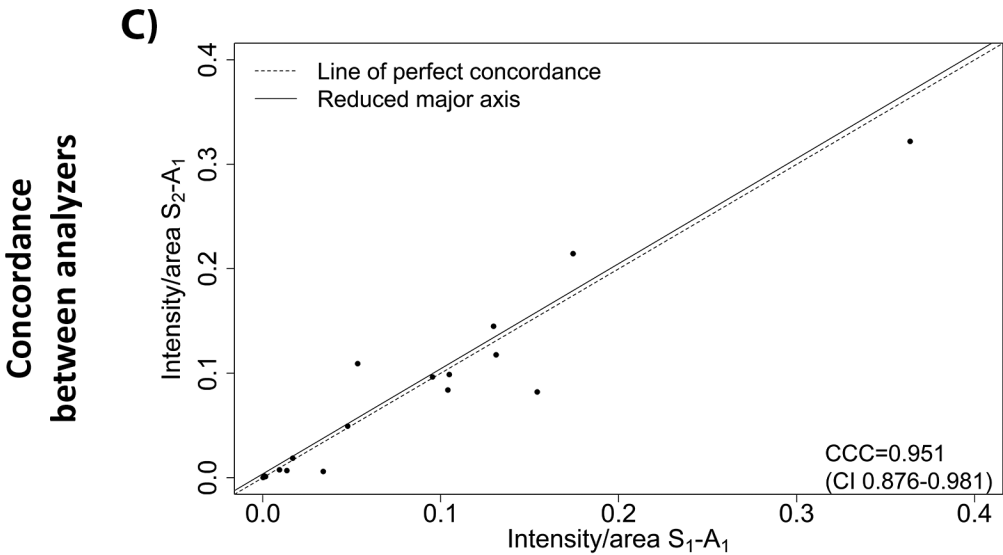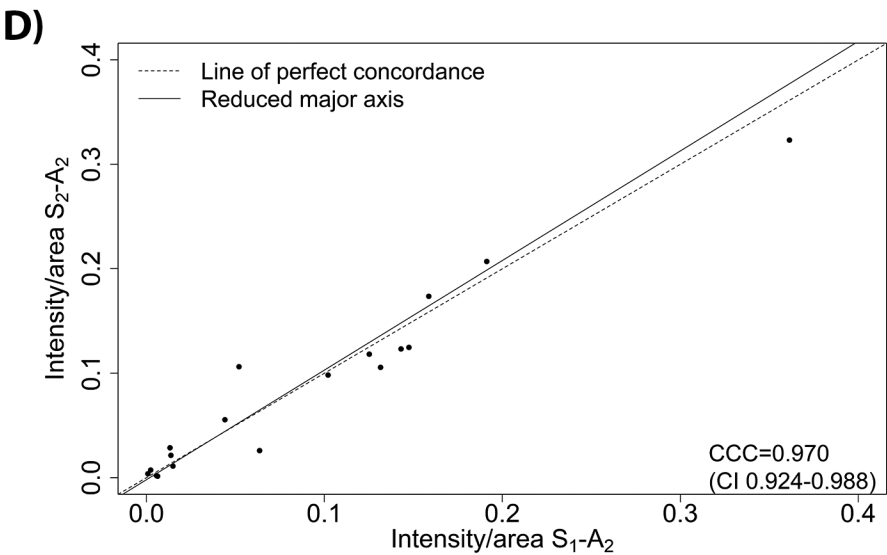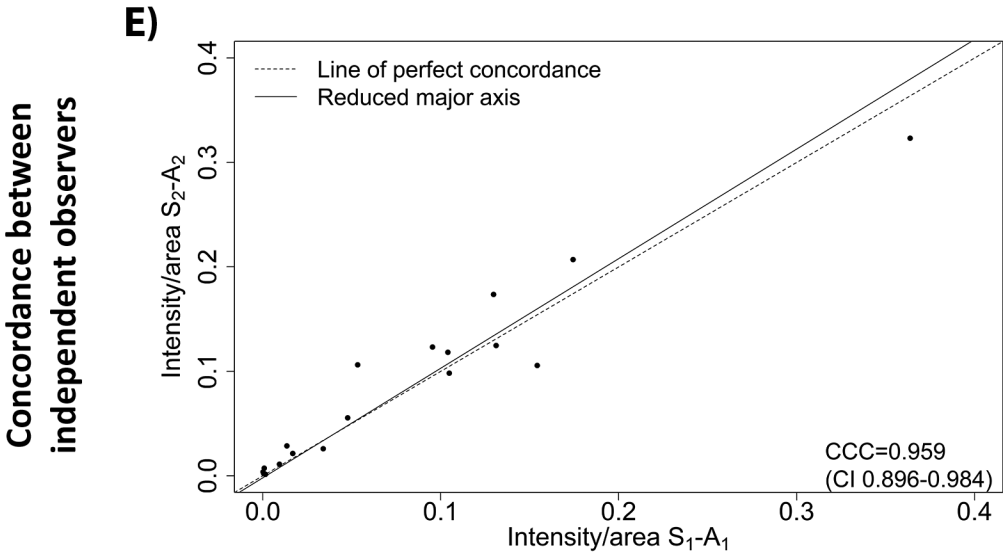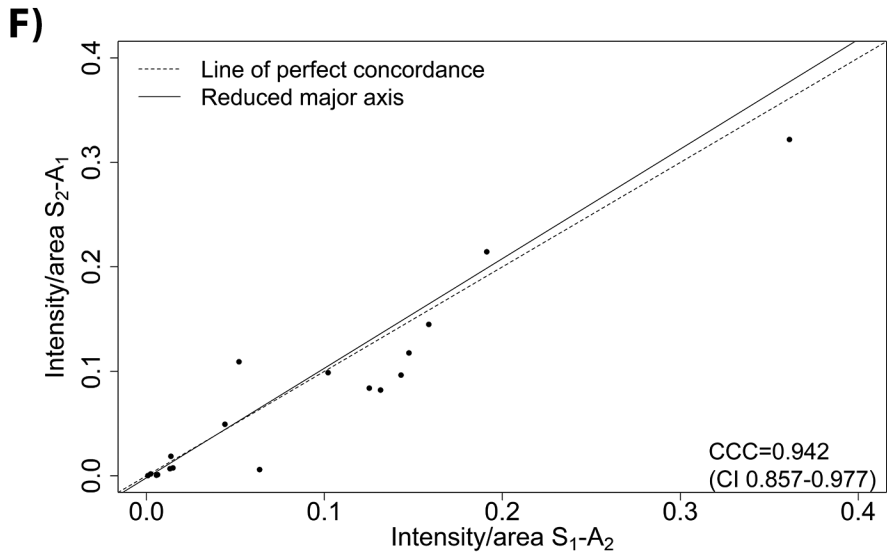

Supplement: Supplementary Figure 3 [file supplementary_figure_3.pdf]

Supplementary Figure 4.

Intensity/area

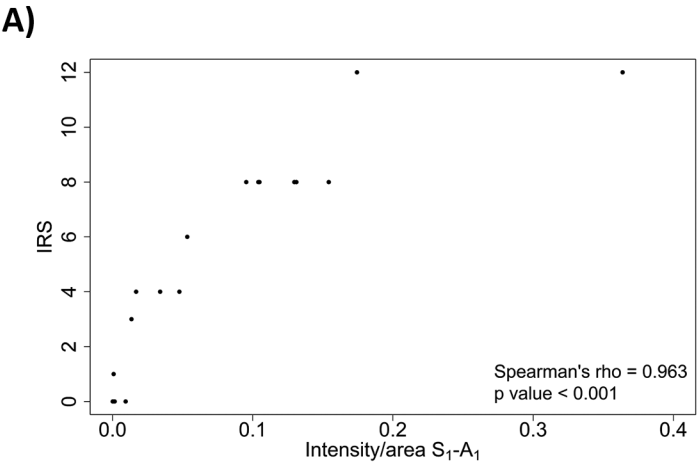

Percentage of positive cells

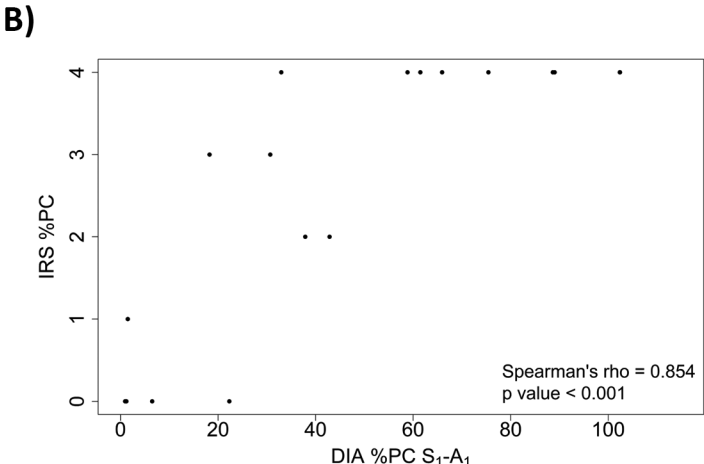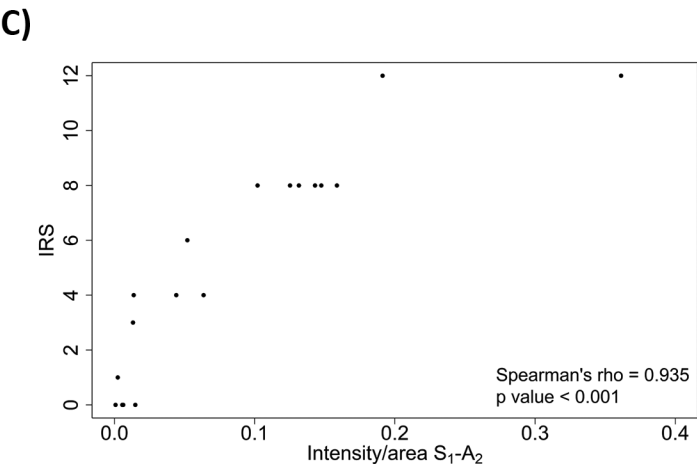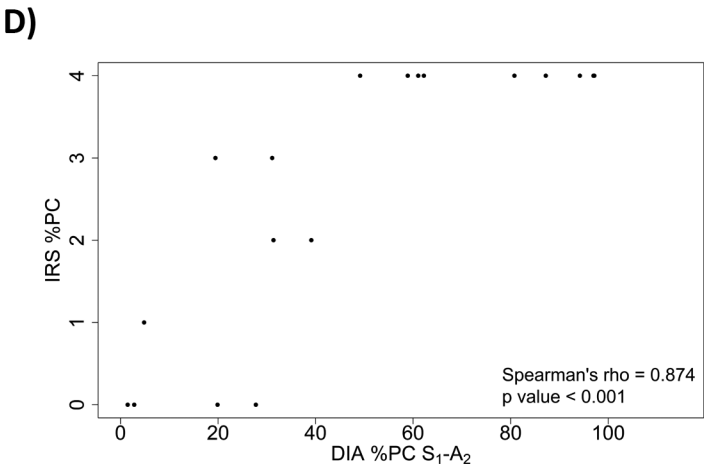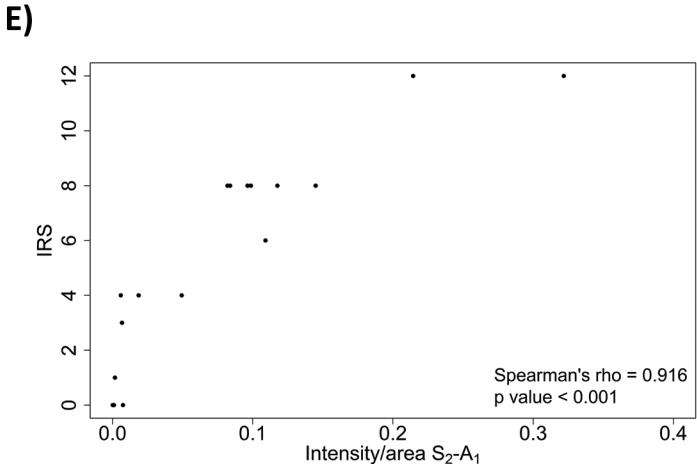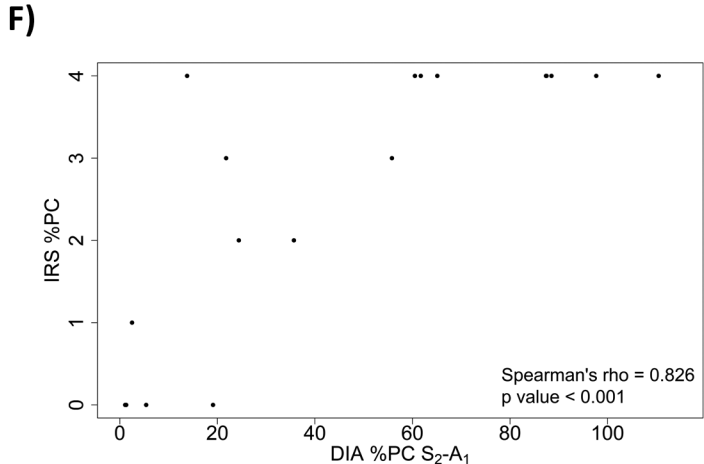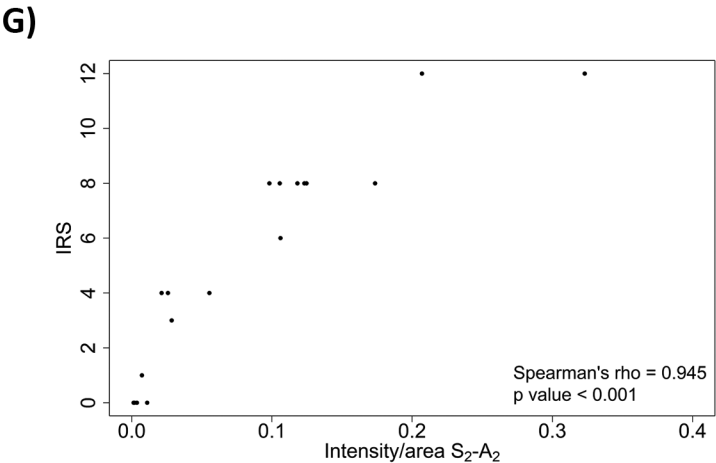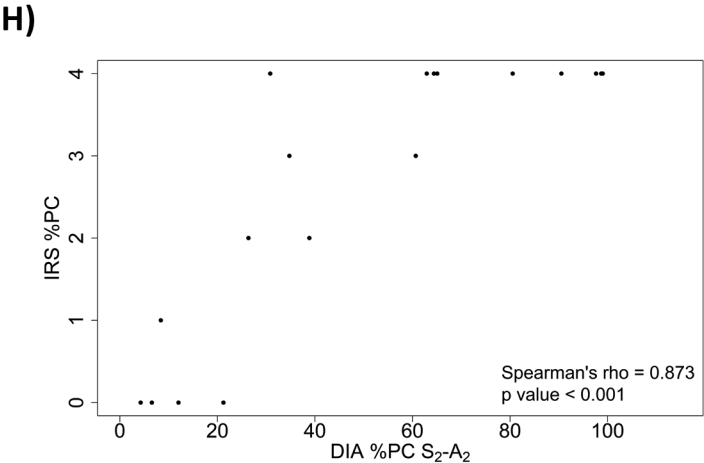

Supplement: Supplementary Figure 4 [file supplementary_figure_4.pdf]

Supplementary Figure 5.

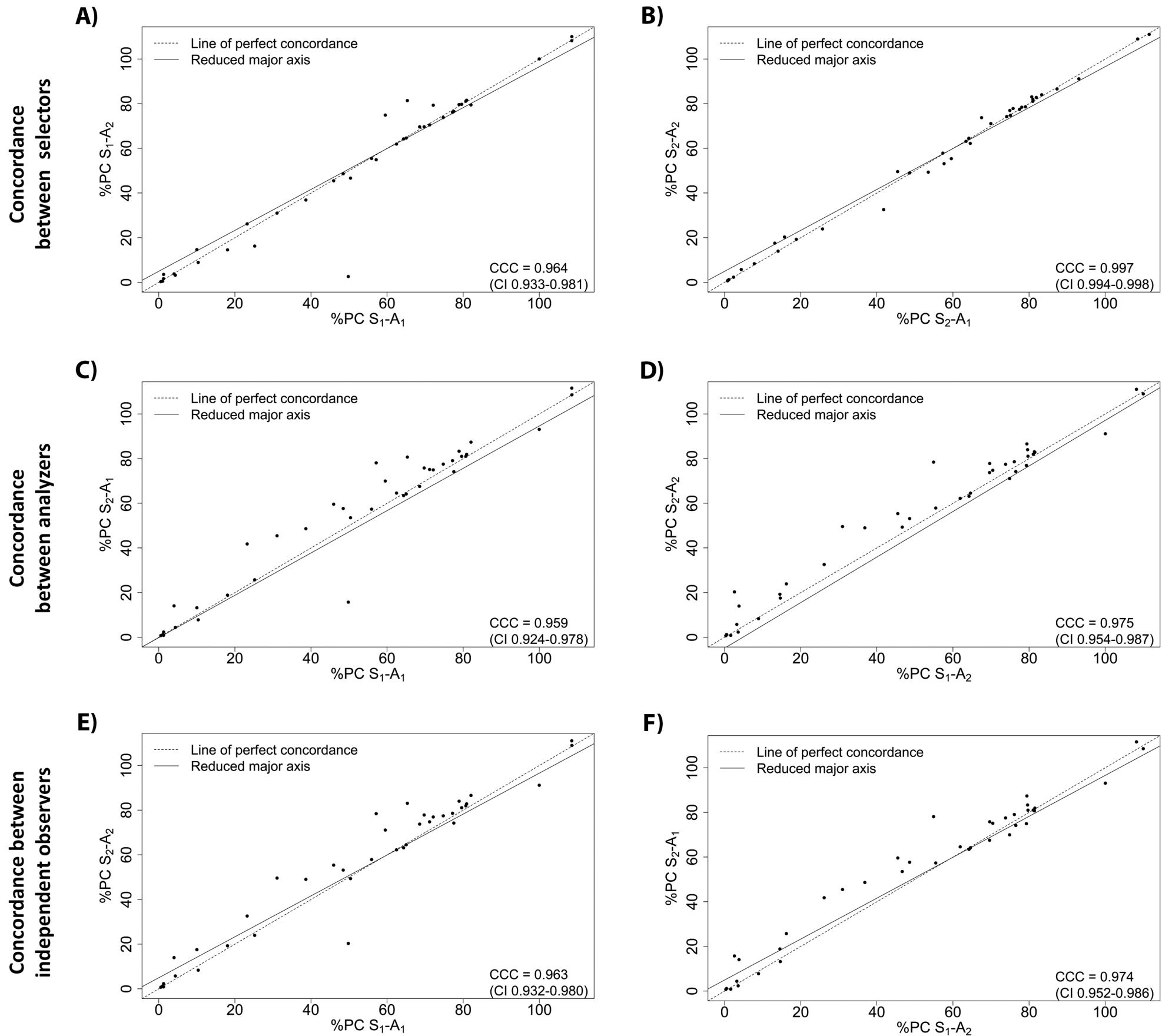

Supplement: Supplementary Figure 5 [file supplementary_figure_5.pdf]

Supplementary Figure 6.

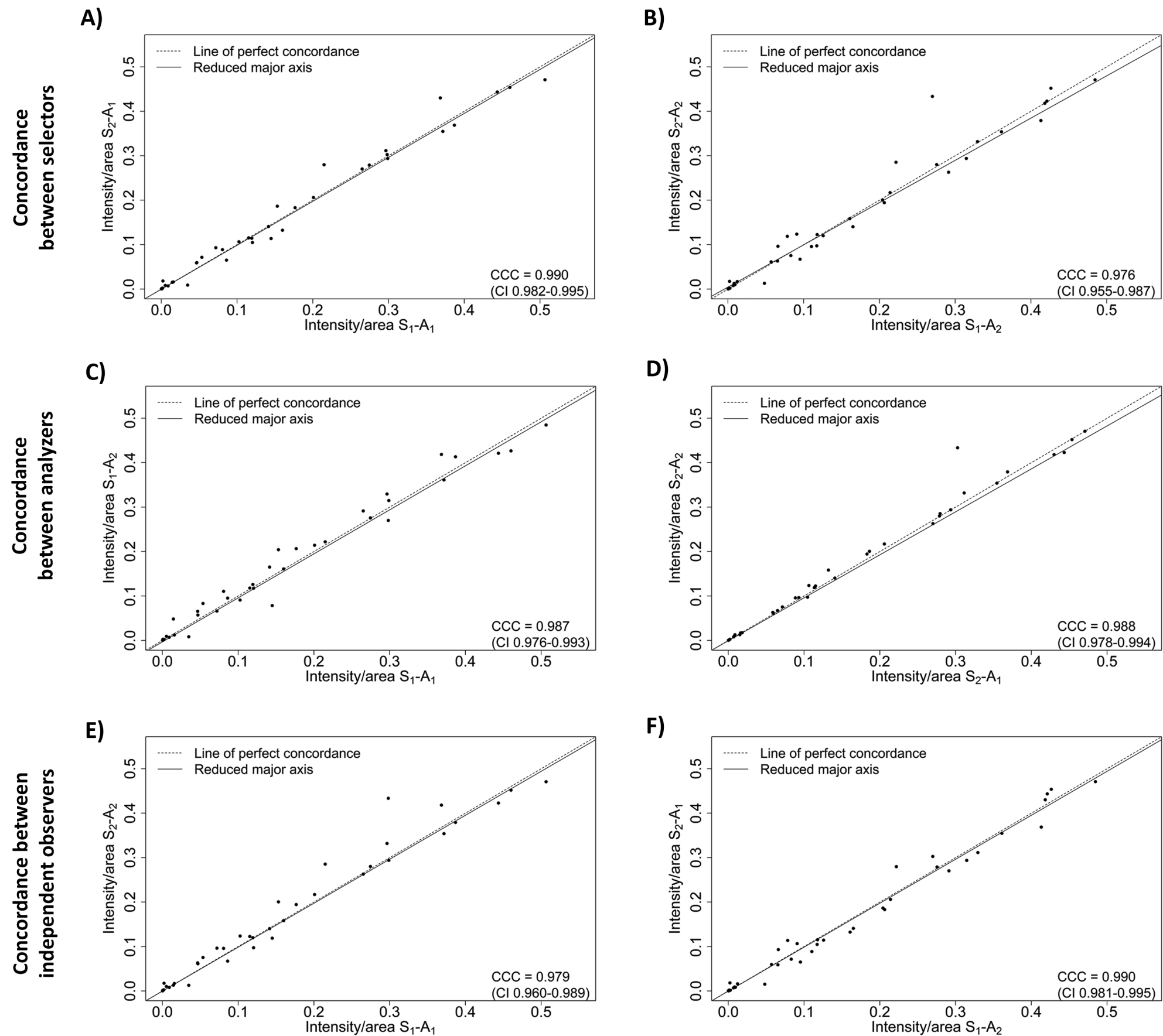

Supplement: Supplementary Figure 6 [file supplementary_figure_6.pdf]

Supplementary Figure 7.

Intensity/area

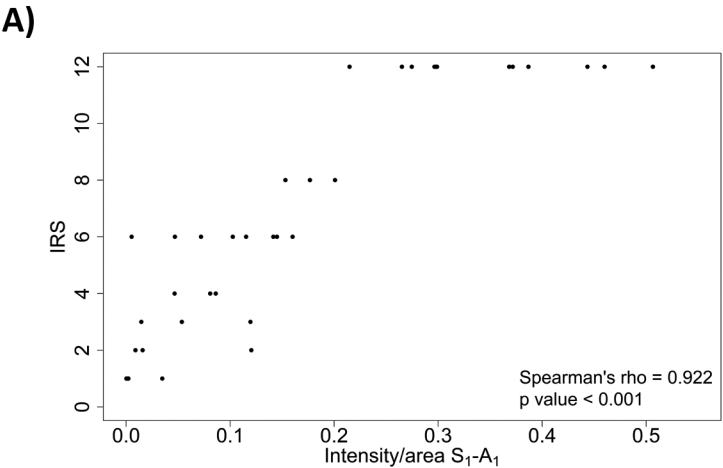

Percentage of positive cells

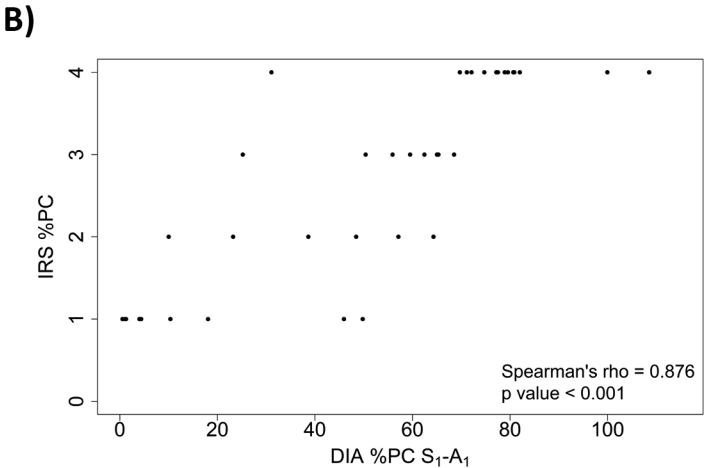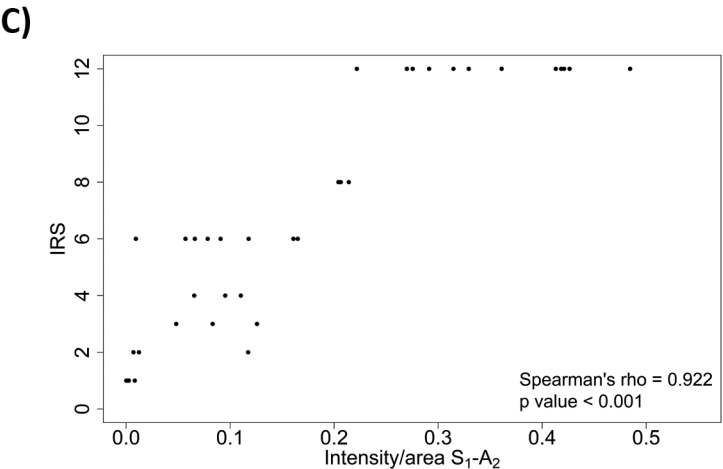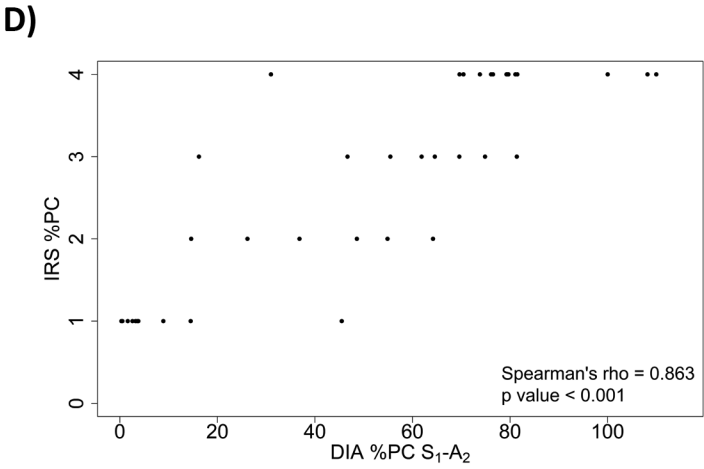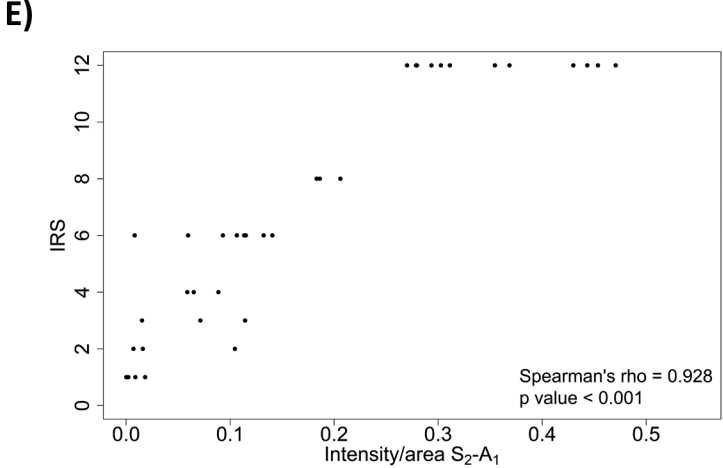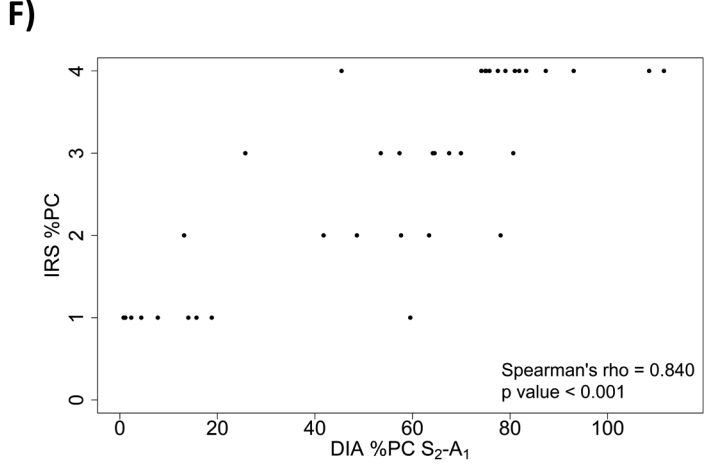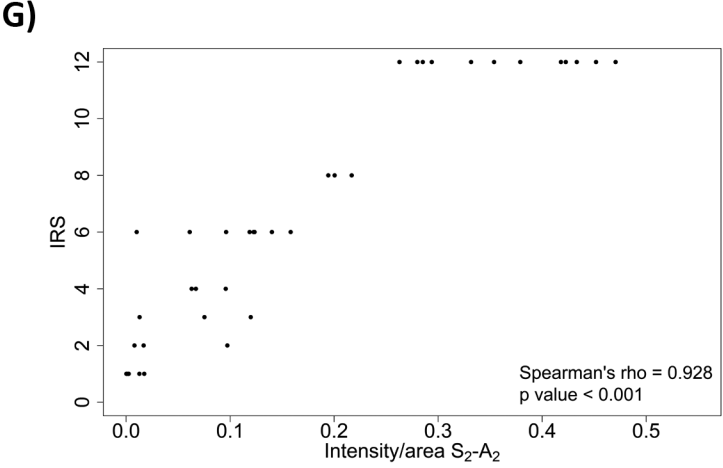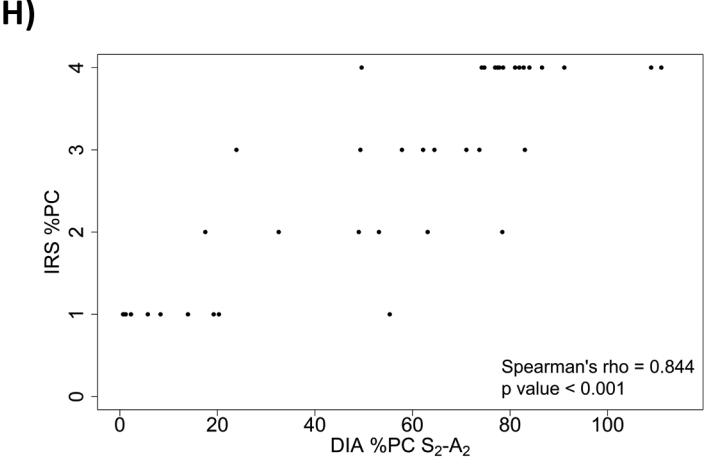

Supplement: Supplementary Figure 7 [file supplementary_figure_7.pdf]

Supplementary Figure 8.

Intensity/area

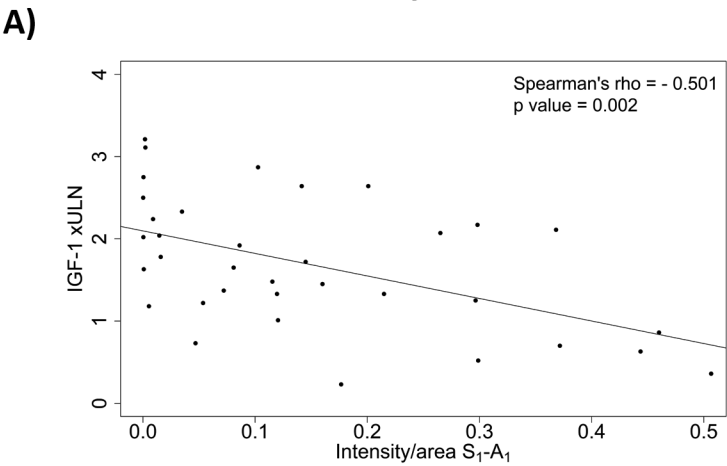

Percentage of positive cells

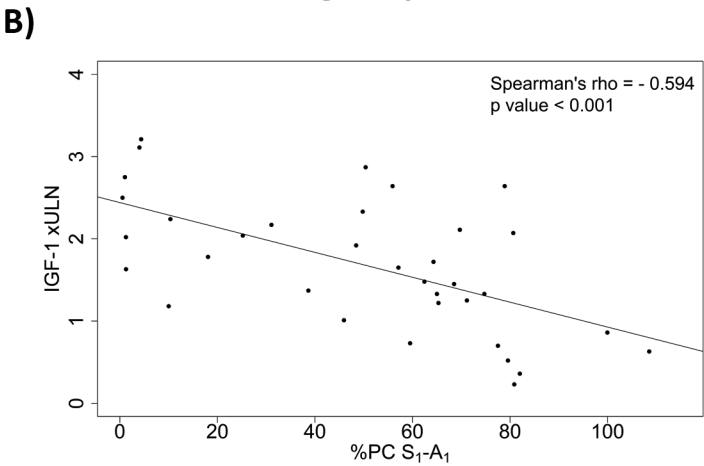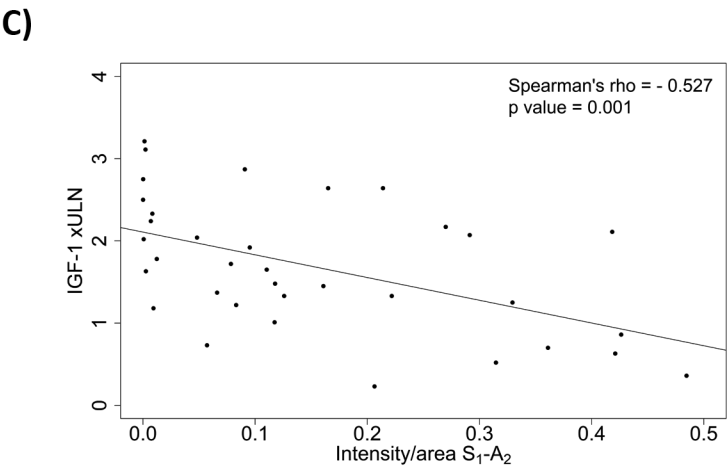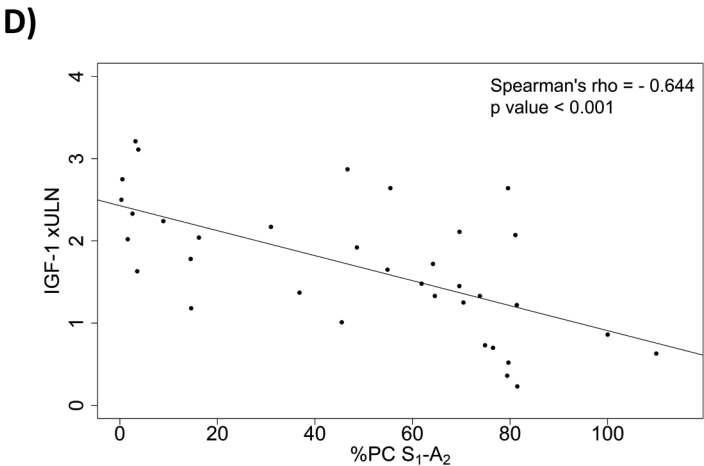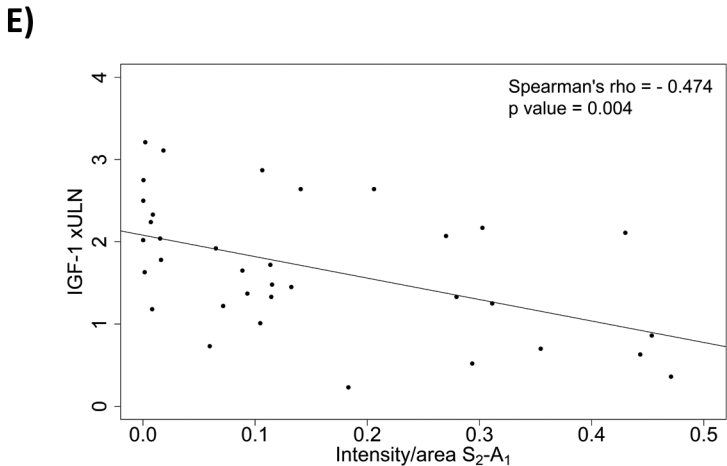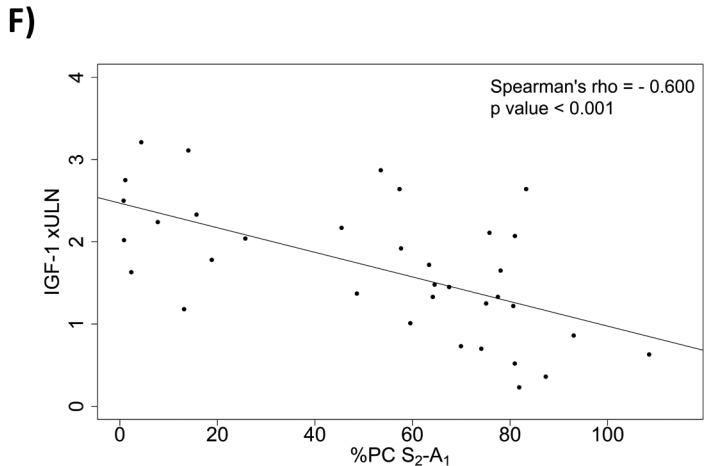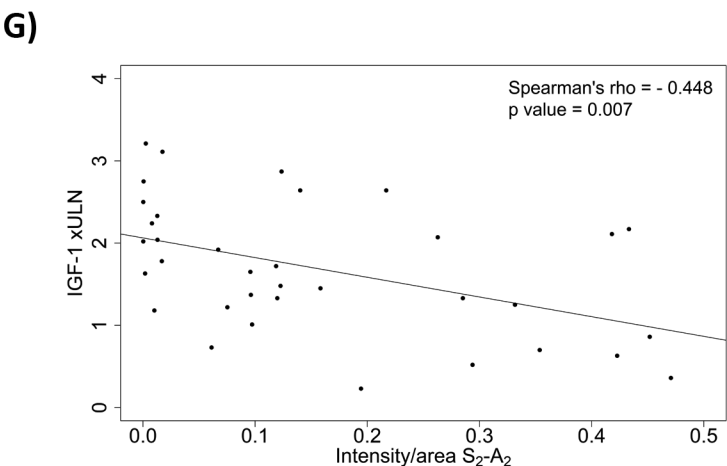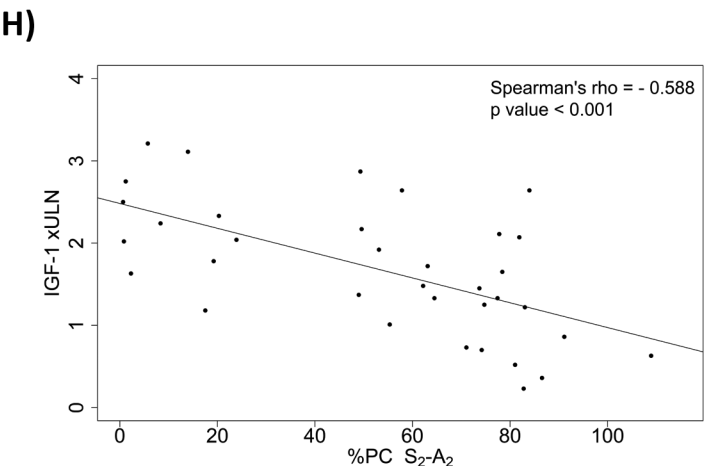

Supplement: Supplementary Figure 8 [file supplementary_figure_8.pdf]
